# Comparison of infectious agents detected from hatchery and wild juvenile Coho salmon in British Columbia, 2008-2018

**DOI:** 10.1371/journal.pone.0221956

**Published:** 2019-09-03

**Authors:** Omid Nekouei, Raphael Vanderstichel, Karia H. Kaukinen, Krishna Thakur, Tobi Ming, David A. Patterson, Marc Trudel, Chrys Neville, Kristina M. Miller

**Affiliations:** 1 Department of Health Management, University of Prince Edward Island, Charlottetown, PE, Canada; 2 Pacific Biological Station, Fisheries and Oceans Canada, Nanaimo, BC, Canada; 3 Fisheries and Oceans Canada, Cooperative Resource Management Institute, School of Resources and Environment Management, Simon Fraser University, Burnaby, BC, Canada; 4 St. Andrews Biological Station, Fisheries and Oceans Canada, St. Andrews, NB, Canada; 5 Forest and Conservation Sciences, University of British Columbia, Vancouver, BC, Canada; Swedish University of Agricultural Science, SWEDEN

## Abstract

Infectious diseases are potential contributors to decline in Coho salmon (*Oncorhynchus kisutch*) populations. Although pathogens are theoretically considered to pose higher risk in high-density rearing environments like hatcheries, there is no direct evidence that hatchery-origin Coho salmon increase the transmission of infectious agents to sympatric wild populations. This study was undertaken to compare prevalence, burden, and diversity of infectious agents between hatchery-reared and wild juvenile Coho salmon in British Columbia (BC), Canada. In total, 2,655 juvenile Coho salmon were collected between 2008 and 2018 from four regions of freshwater and saltwater in BC. High-throughput microfluidics qPCR was employed for simultaneous detection of 36 infectious agents from mixed-tissue samples (gill, brain, heart, liver, and kidney). Thirty-one agents were detected at least once, including ten with prevalence >5%. *Candidatus Brachiomonas cysticola*, *Paraneuclospora theridion*, and *Parvicapsula pseudobranchiocola* were the most prevalent agents. Diversity and burden of infectious agents were substantially higher in marine environment than in freshwater. In Mainland BC, infectious burden and diversity were significantly lower in hatchery smolts than in wild counterparts, whereas in other regions, there were no significant differences. Observed differences in freshwater were predominantly driven by three parasites, *Loma salmonae*, *Myxobolus arcticus*, and *Parvicapsula kabatai*. In saltwater, there were no consistent differences in agent prevalence between hatchery and wild fish shared among the west and east coasts of Vancouver Island. Although some agents showed differential infectious patterns between regions, annual variations likely contributed to this signal. Our findings do not support the hypothesis that hatchery smolts carry higher burdens of infectious agents than conspecific wild fish, reducing the potential risk of transfer to wild smolts at this life stage. Moreover, we provide a baseline of infectious agents in juvenile Coho salmon that will be used in future research and modeling potential correlations between infectious profiles and marine survival.

## Introduction

Coho salmon (*Oncorhynchus kisutch*) is one of seven Pacific salmon species with cultural, recreational, and economic significance to the residents of the North East Pacific region, especially in Canada [1]. Distinct populations of Coho salmon are found in the coastal streams and rivers of British Columbia (BC) and most of them have a three-year lifecycle [1,2]. Wild Coho salmon populations in the United States and Canada have been in decline for over three decades [3–5]. For instance, in the Interior Fraser River watershed, the abundance of Coho salmon have decreased by more than 60% since 1996, with marine survival reaching an all-time low of 0.1–0.3% in 2014 [2]. Returns for some of Coho salmon populations are so low that they have been listed as endangered or threatened by the Committee on the Status of Endangered Wildlife in Canada or through the Endangered Species Act in the United States [2,6]. Several factors are believed to have contributed to population declines, including climate change, food availability in the marine environment, infectious diseases, predation, fishing, land-use activities, and synergistic effects among these factors [4,5,7].

To increase the abundance of Coho salmon and enhance fishing opportunities along the coast of Canada, the Salmonid Enhancement Program was established in 1970s by Fisheries and Oceans Canada (DFO) and hatchery production of these species was initiated [8]. Hatcheries were considered to be effective because the egg-to-smolt survival in hatcheries was significantly higher than that in wild stocks [9]. However, in the marine environment, lower survival of hatchery-origin fish compared to wild fish has been reported [10]. It has been suggested that due to domestication, hatchery fish generally show reduced swimming ability [11] and lower resistance to stress and diseases than their wild counterparts [12]. There is even evidence that a single generation of hatchery production can reduce the genetic fitness of wild fish [13,14]. Given these findings, the continued use of enhancement hatcheries to produce large numbers of fish for exploitation has been debated. Genetic introgression, overcrowding, competition, predation, predator attraction, and transfer of pathogens and disease are all factors that may carry negative consequences from hatchery to wild fish [15–17]. Although infectious diseases are theoretically considered to pose higher risk in high-density rearing environments like hatcheries, there is still no study showing that hatchery-origin Coho salmon increase the transmission of infectious agents to sympatric wild populations [16,18]. Infectious diseases can disrupt salmon’s normal behaviour and physiological performance (e.g. swimming and visual acuity), immunological function, feeding and growth, and can cause mortality in severe cases [7,19]. There is a clear knowledge gap regarding pathogens that can adversely affect the performance and survival of Coho salmon. Out-migrating juveniles are particularly vulnerable to environmental stressors, including infectious agents, during their early marine life, and >90% of them may die in this limited period [4,20,21].

High rearing densities in hatchery environments increase the potential for enhanced transmission of pathogens, but the use of antibiotics and other mitigation measures, such as broodstock selection to minimize vertical transmission of *Renibacterium salmoninarum*, may reduce the incidence and spread of diseases. Alternatively, as many hatcheries use ground rather than river water, hatchery fish may be less exposed to myxozoan parasites that have an alternate invertebrate host in natural freshwater systems. Previous research by our group suggested that naturally occurring myxozoan parasites may be a risk for wild salmon in the ocean [22,23]. Given the observed lower survival of hatchery fish compared to wild fish in the ocean [4], if infection is driving this difference, we would expect that hatchery fish be more vulnerable to infection. As such, we undertook the present cross-sectional study to test the hypothesis that hatchery-reared Coho salmon smolts carry a higher burden of infectious agents at the time they are released from the hatchery compared to their wild counterparts, and that they continue to carry higher agent burdens in the early marine environment. To test this hypothesis, we applied a high throughput microfluidics system to detect and quantitate 36 infectious agents in juvenile Coho salmon sampled in BC, and compared the prevalence, diversity, and overall infection burden of detected agents between hatchery-origin and wild fish over the last 11 years (2008–2018).

## Materials and methods

The animal care and use protocol for this work was approved by the DFO (Fisheries and Oceans Canada) Pacific Region Animal Care Committee (Animal Use Protocol Number: 13–008).

### Sample collection

Sampling of juvenile Coho salmon was carried out between 2008 and 2018 (11 year-classes), over the first nine months of early migration. In freshwater, sampling of wild fish was carried out using beach seining within natal lakes or dip netting at smolt fences, generally before hatchery releases. In hatcheries, fish were sampled just prior to release. In the marine environment, fish were collected via mid-water trawl sampling from the Canadian Coast Guard vessel, WE Ricker, or by trawl or purse seine sampling from smaller fishing vessels contracted by DFO. On the trawl vessels, fish were kept on ice and processed within 30 minutes of being brought on-deck. On purse seine vessels, samples were placed in a holding tank on deck until processed. In general, all fish propagated artificially from eggs and milt from the group of spawners and maintained in a controlled hatchery environment until their release are defined as “hatchery fish”. Wild fish are defined as fish that developed from eggs produced by parents that spawned naturally in the river bed. In the marine environment, hatchery-origin and wild juvenile Coho salmon were identified as marked and unmarked (with and/or without adipose fin clips) by visual assessment, and/or fish that carried a coded wire tag (CWT). While all clipped and/or CWT fish were known hatchery-origin, there was a slight chance of misclassifying hatchery as wild fish due to different reasons, such as failure in marking, partial clips, or the wire tag was not readily detectable. In addition, in systems whereby hatcheries release fry into lakes (as fry are too small to clip or tag), these would be indistinguishable from wild salmon. However, since these fish rear naturally, we would expect them to closely resemble wild fish with respect to their potential exposure to infectious agents.

**Fig 1:**
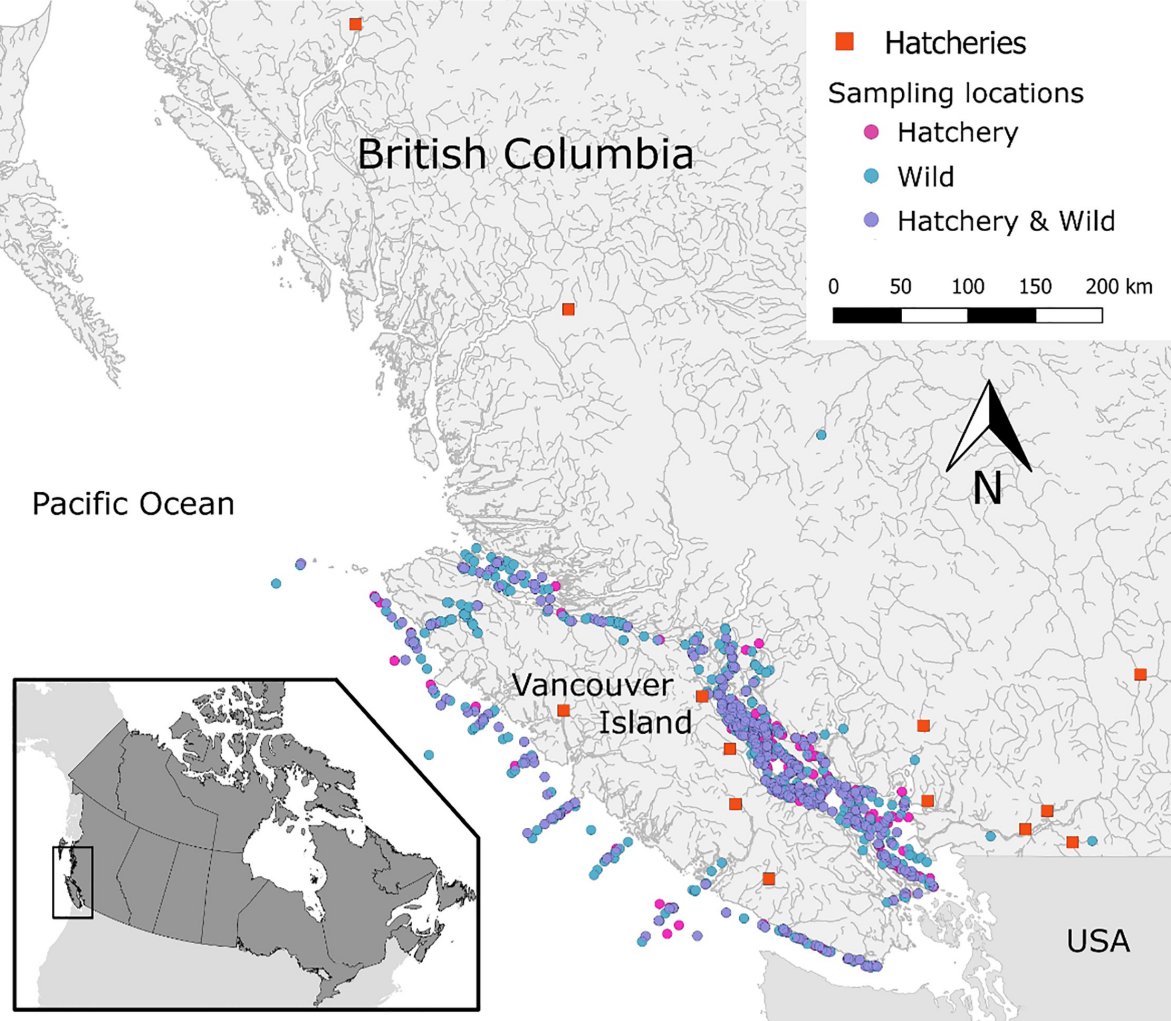
Map of British Columbia, Canada, illustrating sampling locations for 2,655 juvenile Coho salmon by their origin (hatchery based or wild), between 2008 and 2018. This map was created in QGIS v2.18.13 (http://www.qgis.org).

Upon sampling, live fish were euthanized in MS-222, assigned a unique identification code, and five tissues (gill, whole brain, heart, liver, and kidney) were sampled aseptically from each fish and preserved in RNAlater (Qiagen, MD, USA). Tissue samples in RNAlater were kept at 4°C for 24 hours, and transferred to -20°C for short-term or -80°C for long-term storage until further processing. Additional fish collected at sea and most freshwater collections were frozen in dry ice and moved to -80°C until further processing in the laboratory. Overall, 2,655 juvenile Coho were used in our study, including fish sampled in freshwater drainages in the BC Mainland (i.e. Fraser River watershed and central coast) and Vancouver Island, and marine samples within the Salish Sea on the inside waterway between Vancouver Island and the Mainland, and on the west coast of Vancouver Island (Fig 1).

### Laboratory analysis

The laboratory methods presented herein have been summarized/adapted from a recent publication by the corresponding author of the present manuscript [24].

Sampled tissues were individually homogenized in TRI-reagent (Ambion Inc., Austin, TX, USA) before extraction. ‘1-bromo-3-chloropropane’ was added to the homogenate, which was then centrifuged to separate aqueous and organic phases. Equal volumes of the aqueous phase for each tissue within a fish were combined and placed into a 96-well plate for RNA extraction. Total RNA was extracted from each sample using the MagMax-96 for Microarrays Total RNA Isolation Kit (Ambion Inc.) on a Biomek NXP automated liquid-handling instrument using the ‘spin method’ protocol. RNA quantity and quality were assessed by measuring the A260/280/230 on a Beckman Coulter DTX 880 Multimode Detector (Brea, CA, USA), and samples diluted to 62.5 ng/μL. RNA (1 μg) was reverse-transcribed to cDNA using SuperScript VILO MasterMix (Invitrogen, Carlsbad, CA) as per the manufacturer’s protocol.

DNA was isolated from the organic/interphase portion of TRI-reagent using a high salt TNES-urea buffer [25], followed by the BioSprint 96 DNA extraction kit on a BioSprint 96 workstation (Qiagen, MD). DNA quantity and quality were assessed by measuring the A260/280 on a Beckman Coulter DTX 880 Multimode Detector (Brea, CA, USA), and normalized to 62.5 ng/μL. Equal volumes of DNA and cDNA were combined and used as template for the qPCR assays.

Combined multi-tissue samples of DNA/cDNA for each fish were assessed for the presence and load (abundance) of our infectious agents of interest. Assays were run in duplicate on the Fluidigm BioMark^TM^ HD platform (Fluidigm, South San Francisco). The platform capacity is 96 assays on 96 samples at once (9,216 individual reaction chambers). Assays assessed within the current study included 36 agents in duplicate, and one housekeeping gene (endogenous control to assess RNA quality). The assays included 18 parasites, 12 bacteria, and 6 viruses (Table 1).

**Table 1:** Thirty-six infectious agents tested on 2,655 juvenile Coho salmon, between 2008 and 2018, along with their overall prevalences (%). The 31 detected agents are presented in the decreasing order of prevalence.

Agent	Abbreviation	Type ^a^	N	Positive	Prevalence ^b^
**1**. *Candidatus Brachiomonas cysticola*	c_b_cys	B	2,647	2,363	89.3
**2**. *Paraneuclospora theridion*	pa_ther	P	2,596	1,023	39.4
***3***. *Parvicapsula pseudobranchiocola*	pa_pse	P	2,584	965	37.3
**4**. *Loma salmonae*	lo_sal	P	2,624	756	28.8
**5**. *Parvicapsula minibicornis*	pa_min	P	2,596	691	26.6
**6**. *Myxobolus arcticus*	my_arc	P	2,640	289	10.9
**7**. *Flavobacterium psychrophilum*	fl_psy	B	2,623	217	8.3
**8**. 1. Gill chlamydia	sch	B	2,638	202	7.7
**9**. *Parvicapsula kabatai*	pa_kab	P	2,645	164	6.2
**10**. *Ceratonova shasta*	ce_sha	P	2,646	144	5.4
**11**. *Tetracapsuloides bryosalmonae*	te_bry	P	2,648	129	4.9
**12**. *Ichthyophonus hoferi*	ic_hof	P	2,644	97	3.7
**13**. Erythrocytic necrosis virus	env	V	2,641	91	3.4
**14**. *Tenacibaculum maritimum*	te_mar	B	2,622	85	3.2
**15**. Piscine OrthoReovirus	prv	V	2,652	79	3.0
**16**. *Sphaerothecum destructuens*	sp_des	P	2,653	55	2.1
**17**. *Myxobolus insidiosus*	my_ins	P	2,651	49	1.8
**18**. *Nanophyetus salmincola*	na_sal	P	2,654	48	1.8
**19**. *Crybtobia salmonistica*	cr_sal	P	2,655	43	1.6
**20**. *Kudoa thyrsites*	ku_thy	P	2,649	45	1.7
**21**. *Facilispora margolisi*	fa_mar	P	2,626	36	1.4
**22**. *Ichthyophthirius multifiliis*	ic_mul	P	2,571	21	0.8
**23**. *Renibacterium salmoninarum*	re_sal	B	2,654	16	0.6
**24**. Viral hemorrhagic septicemia virus	vhsv	V	2,640	18	0.7
**25**. *Dermocystidium salmonis*	de_sal	P	2,655	17	0.6
**26**. *Piscichlamydia salmonis*	pch_sal	B	2,569	16	0.6
**27**. Rickettsia-like organism	rlo	B	2,651	10	0.4
**28**. *Vibrio salmonicida*	vi_sal	B	2,650	6	0.2
**29**. *Neoparamoeba perurans*	ne_per	P	2,654	5	0.2
**30**. *Piscirickettsia salmonis*	pisck_sal	B	2,650	4	0.2
**31**. Pacific salmon parvovirus	pspv	V	2,651	4	0.2
**32**. *Yersinia ruckeri*	ye_ruc_glnA	B	2,654	1	0.0
**33**. *Aeromonas salmonicida*	ae_sal	B	2,655	0	0.0
**34**. Infectious hematopoietic necrosis virus	ihnv	V	2,655	0	0.0
**35**. Viral encephalopathy & retinopathy virus	ver	V	2,655	0	0.0
**36**. *Vibrio anguillarum*	vi_ang	B	2,655	0	0.0

^a^Type of agent: B = Bacterium, V = Virus, P = Parasite

^b^Overall ‘prevalence’ was defined as the number of test-positive samples divided by the total number of samples tested for each given infectious agent with conclusive results (i.e. positive / N).

On each dynamic array, there were 80 samples and 16 controls run. Negative controls included two negative processing controls for RNA/DNA extraction, two no-template controls for template enrichment (described below), two cDNA (no reverse transcriptase) controls, and two no-template controls for PCR. Positive controls included duplicates of a pooled sample from the cDNA/DNA for all fish used in the study and six serial dilutions of APC clones for all assays to both assess assay integrity and to calculate the copy number of each detected agent; APC clones were loaded last to minimize the potential for contamination. The APC clones were synthesized and cloned sequences of the amplicon for each assay contained an “extra” probe sequence so that potential contamination of high concentration APCs in sample wells could be identified.

BioMark microfluidics qPCR was performed as described in Miller et al. (2016) [26]. Briefly, minute assay volumes (7 nL) are used; therefore, necessitating a pre-amplification step of assays to optimize sensitivity. Dilute primer pairs for each of the 48 assays were combined with TaqMan Preamp MasterMix (Applied Biosystems, Foster City, California) for a final concentration of 50 nM in a 5 μL reaction, and run through 14 cycles of amplification, according to the BioMark protocol. ExoSAP-IT (Affymetrix, Santa Clara, CA) was used to remove unincorporated primers before the samples were diluted 1:5 in DNA Suspension Buffer (Teknova, Hollister, CA).

A 5 μL sample mix was prepared for each pre-amplified sample with TaqMan Universal Master Mix (Life Technologies), GE Sample Loading Reagent (Fluidigm), and a 5 μL aliquot of assay mix was prepared containing 10 μM primers and 3 μM probes for each separate TaqMan assay. An IFC controller HX pressurized and mixed the assays and samples from their individual inlets on the chip. PCR conditions were: 50°C for 2 min, 95°C for 10 min, followed by 40 cycles of 95°C for 15 s, and 60°C for 1 min on the BioMark Dynamic Array. Cycle threshold (Ct) was determined using the BioMark Real-Time PCR analysis software. Visual evaluation identified abnormal curve shapes, presence of APC contamination, and correlation of replicates.

For each infectious agent assay in this study, the analytically validated limit of detection (LOD) [26] was applied to the average Ct values (from duplicate qPCR assays) to categorize the test results as either positive or negative. These dichotomized results were further used in the calculation of prevalence and other statistical analyses. The limit of detection is defined as the estimated Ct number under which a given assay is expected to provide true positive results in 95% of the times [26]. If an infectious agent assay detected a Ct signal in only one of the two replicates, the sample was considered ‘inconclusive’ for that assay and treated as a missing value in our final analyses. While this platform has been proved to provide reliable, rapid, and inexpensive quantitative data on microbe presence and load, its performance for diagnostic purposes in salmon has not been evaluated; however, it has already been applied for human viral and bacterial diagnostics and water quality testing [26]. As such, calculated prevalence for each infectious agent should be interpreted as the test prevalence for the detection of that agent. Due to the use of mixed-tissue samples (including gill), the detection prevalence may not necessarily be equivalent to the prevalence of systemic infection with a given infectious agent, especially in cases with borderline Ct-values. A formal analytical evaluation of our screening platform for application in microbe surveillance research has been implemented and published in ‘Miller et al., 2016’, including all technical details and analytical validation procedures [26].

### Statistical analysis

The final dataset included 2,655 juvenile Coho salmon. All statistical analyses were carried out in Stata v15.1 (StataCorp, College Station, TX, USA).

To effectively compare infectious agents’ profiles between hatchery-origin and wild fish in our study, sampling locations were categorized into four main geographical regions, including: (1) freshwater Mainland BC, (2) freshwater Vancouver Island (VI), (3) saltwater/marine on the east coast of VI, also known as the Salish Sea, and (4) saltwater/marine on the west coast of VI. These geographic separations represent the distinct environments encountered along the migration path of the fish. Fig 1 displays the localization of captured fish in BC, colored by the origin of fish (hatchery or wild). This map was created using coordinates of the sampling locations in QGIS v2.18.13 (http://www.qgis.org).

The term ‘prevalence’ for each infectious agent was defined as the number of fish with the agent detected (positives) divided by the total number of fish tested with conclusive results for that agent. All of the 46 infectious agents and their overall prevalence of detection are reported in Table 1.

The term ‘diversity’ for each sampled fish was defined as the sum of all infectious agents detected from that sample (i.e. the number of co-detections per fish). A mixed-effects Poisson regression model was built to compare the diversity of infectious agents between hatchery-origin and wild fish within the four sampling regions, including the random effects of year (the outcome of interest was the number of co-detections).

The term ‘relative infection burden’ (RIB) is a composite metric of multiple infectious agent burden using qPCR data, which was calculated from the following formula:

$$\sum_{i\in m}^{m}\frac{Li}{Lmaxi}$$

Where for a given fish, RNA copy number of the i’th positively-detected infectious agent (*Li*) is divided by the maximum RNA copy number within the population for the i’th infectious agent (*Lmaxi*), and then summed across all detected agents from that fish [27]. A mixed-effects linear regression model was built to compare the RIB of agents between hatchery-origin and wild fish within the four sampling regions, including the random effects of year (the outcome of interest was log_10_-RIB).

After determining the overall prevalence of all infectious agents, those with a prevalence > 5% (hereafter, the common agents = the top 10 agents in Table 1) were selected for further statistical analyses. The reason for this selection was to avoid zero counts and/or extremely unbalanced distributions of positive samples (at the eight region×origin combinations).

Mixed-effects logistic regression models were built to compare the prevalence of the common infectious agents between hatchery-origin and wild fish within the four sampling regions. In these analyses, presence or absence of a particular agent within a sample/fish served as the outcome of interest and the random effects of year were included. Bonferroni correction was applied to the significance level (0.05) in multiple comparisons after each model.

## Results

Overall, 1,116 and 1,539 juvenile Coho salmon with hatchery and wild origins (respectively) were captured and analyzed in our study. The names and locations of the 13 freshwater hatcheries sampled in this study are indicated in S1 Table. Of 2,655 samples, 23.5% were from freshwater and 76.5% from saltwater. The frequency distribution of the sampled fish by sampling region, origin, and year-class is presented in Table 2. As shown in this table, freshwater sampling was not carried out from hatchery and wild fish in all years.

**Table 2:** Frequency distribution of 2,655 collected juvenile Coho salmon samples, by sampling region, year-class, and origin (H: hatchery or W: wild). Four sampling regions: 1) freshwater-Mainland; 2) freshwater-Vancouver Island (VI); 3) saltwater-east coast of VI; and 4) saltwater-west coast of VI.

	FW Main		FW VI		SW East		SW West		
Year	H	W	H	W	H	W	H	W	Total
2008	- ^a^	30	8	-	-	4	-	9	51
2009	-	14	-	-	9	6	13	57	99
2010	-	-	-	-	21	78	26	96	221
2011	-	3	-	-	50	78	14	50	195
2012	-	-	-	-	10	54	10	61	135
2013	130	-	156	-	70	128	35	68	587
2014	124	5	-	36	143	203	10	20	541
2015	-	39	18	31	43	125	49	45	350
2016	-	31	-	-	125	118	1	2	277
2017	-	-	-	-	20	62	-	-	82
2018	-	-	-	-	31	86	-	-	117
Total	254	122	182	67	522	942	158	408	2,655

^a^ dash indicates no sampling event.

Of the 36 infectious agents investigated within each individual fish sample, 31 were detected at least once (Table 1). Ten infectious agents had an overall prevalence > 5% (i.e. the common agents). *Candidatus Brachiomonas cysticola* (c_b_cys) was observed at the highest overall prevalence (89.3%), detected in 2,363 out of 2,647 samples with conclusive results, followed by *Paraneuclospora theridion* (pa_ther, also known as *Desmozoon lepeophtherii*; 39.4%) and *Parvicapsula pseudobranchiocola* (pa_pse; 37.3%). Five infectious agents were not detected at all in our samples. To improve clarity in visualization, only abbreviations for the common infectious agents are used in the Figs (see key in Table 1).

### Diversity

**Fig 2:**
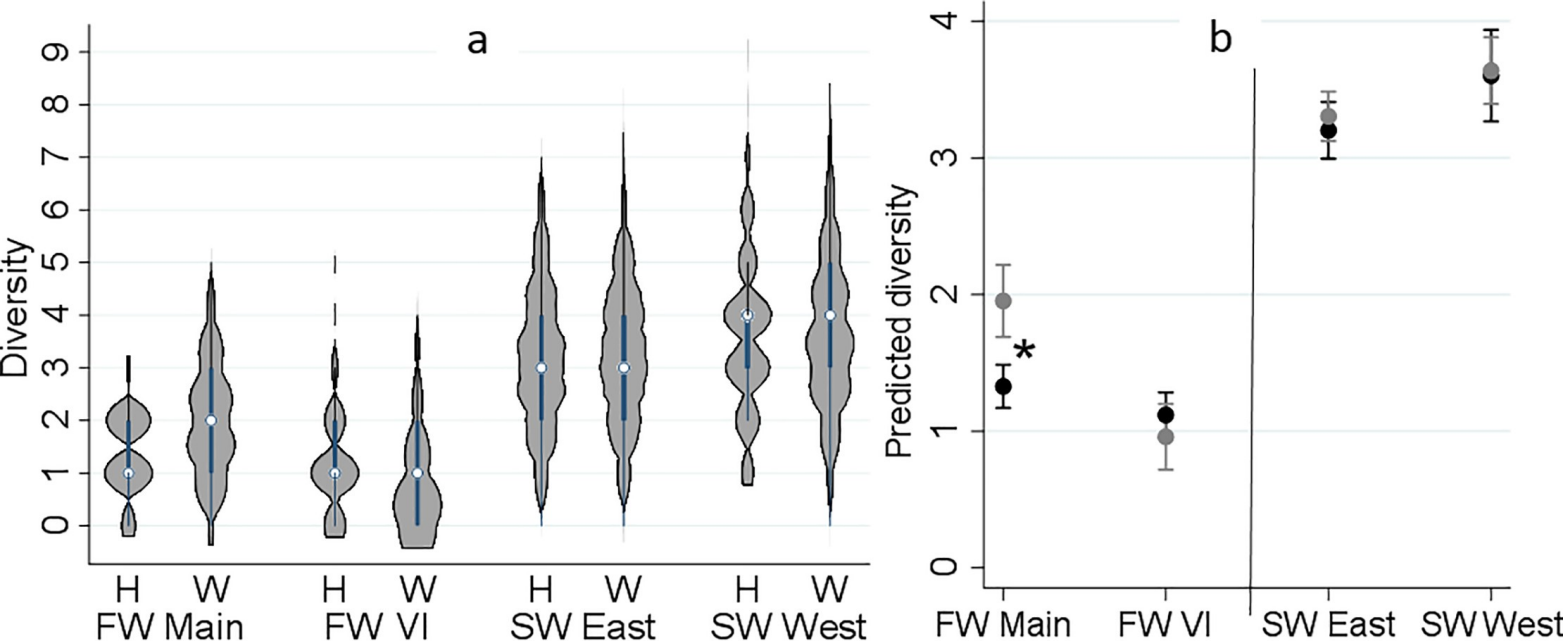
**Distribution of the diversity of infectious agents detected in 2,655 juvenile Coho salmon by sampling region and origin (H: hatchery and W: wild) on left side, and the interaction plot for the results of the Poisson regression model, indicating predicted number of detected infectious agents (Y axis) by sampling region and origin (black: hatchery and grey: wild) on right side. Sampling regions: 1) freshwater-Mainland; 2) freshwater-Vancouver Island (VI); 3) saltwater-east coast of VI; and 4) saltwater-west coast of VI.** *Statistical comparisons (Bonferroni groups) for predicted diversity between hatchery and wild were only significant in freshwater-Mainland (P < 0.001).

The frequency distribution of the diversity of detected agents (co-detections) from each fish by sampling region and origin is presented in Fig 2A. Diversity ranged between 0 and 9, with a median of 3. Approximately, 95% of the samples had a diversity of ≤ 5. The results of the respective Poisson model (indicating changes in the predicted number of detected agents by study region and origin) are illustrated in Fig 2B. The diversity of agents from freshwater to saltwater increased substantially. Hatchery and wild fish did not show any significant difference in diversity, except in freshwater samples from Mainland BC (Fig 2B). In the latter region, wild fish had a significantly greater diversity of agents than hatchery fish (P < 0.001). The variability in diversity between study years was highly significant (P < 0.001).

### Relative infection burden

**Fig 3:**
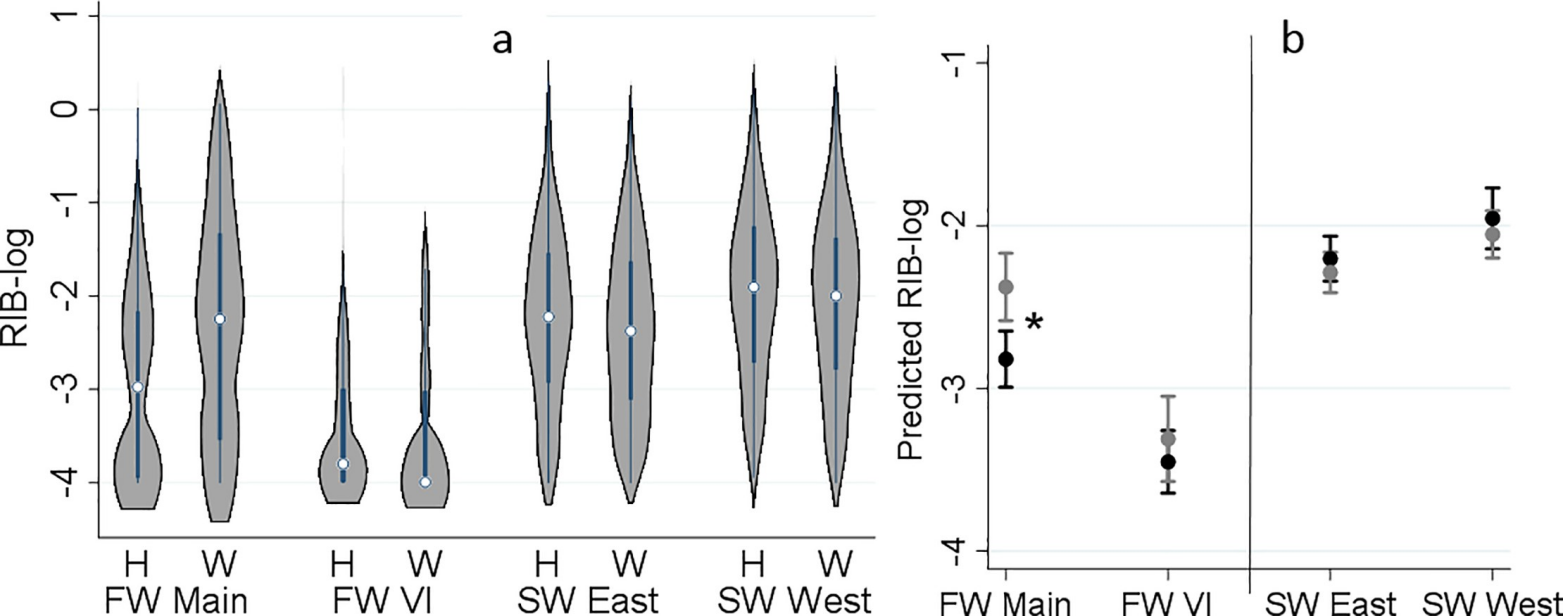
**Distribution of log_10_-relative infection burden (RIB) of all detected infectious agents in 2,655 juvenile Coho salmon by sampling region and origin (H: hatchery and W: wild) on left side, and the interaction plot for the results of the linear regression model, indicating predicted log_10_-RIB (Y axis) by sampling region and origin (black: hatchery and grey: wild) on right side. Sampling regions: 1) freshwater-Mainland; 2) freshwater-Vancouver Island (VI); 3) saltwater-east coast of VI; and 4) saltwater-west coast of VI.** *Statistical comparisons (Bonferroni groups) for predicted RIB-log between hatchery and wild were only significant in freshwater-Mainland (P < 0.001).

RIB ranged between 0 and 2.06, with an extremely right-skewed distribution; therefore, a logarithmic transformation was applied to RIB values. The frequency distribution of log_10_-RIB for detected agents by sampling region and origin is presented in Fig 3A. Consistent with the diversity, a significant increase in RIB was observed from freshwater to saltwater. Moreover, RIB was significantly higher in wild than hatchery fish (P = 0.004) only in freshwater samples from Mainland BC (Fig 3B).

### Prevalence of the common agents

Distribution of the prevalences of the common agents by region and origin are presented in Table 3. Among the ten common agents, the following were detected in both freshwater and marine environments: Ca. *B*. *cysticola*, *Loma salmonae* (lo_sal), *Myxobolus arcticus* (my_arc), and *Parvicapsula kabatai* (pa_kab). *Flavobacterium psychrophilum* (fl_psy) is a known freshwater pathogen and the rest of the prevalent agents were predominantly detected in the marine environment. Due to the scarcity of samples with agent detections for the majority of low-prevalent agents (prevalence <5%; see S2 Table), robust statistical comparison between hatchery and wild fish was not possible; however, the same approach as for the common agents (logistic regression models) was applied for those with a reasonable distribution (1% < prevalence < 5%) in order to provide a general prevalence baseline. Prevalence distributions of the low-prevalent agents are presented in S2 Table. Statistical analyses for the low-prevalent agents did not result in any significant differences in prevalence between hatchery and wild fish (P > 0.05 for all; results not shown).

**Table 3:** Prevalence (%) and the range of samples tested (N) for the prevalent infectious agents from 2,655 juvenile Coho salmon, by sampling region and origin (H: hatchery or W: wild). Four sampling regions: 1) freshwater-Mainland; 2) freshwater-Vancouver Island (VI); 3) saltwater-east coast of VI; and 4) saltwater-west coast of VI. For infectious agents’ complete names, refer to key in Table 1.

Region		Origin	N	c_b_cys	pa_ther	pa_pse	lo_sal	pa_min	my_arc	fl_psy	sch	pa_kab	ce_sha
FW	Main	H	249–254	78.7	0.0	0.0	0.4	0.0	4.3	47.4	0.0	0.0	0.0
		W	120–122	77.9	0.8	0.0	27.3	1.6	31.1	30.6	0.0	6.7	0.0
	VI	H	177–182	62.6	0.0	0.0	12.2	0.0	1.7	10.7	0.0	14.8	0.0
		W	62–67	20.9	0.0	0.0	11.9	0.0	7.5	24.2	0.0	18.2	0.0
SW	East	H	499–521	97.1	44.1	40.8	34.0	41.5	6.2	1.4	12.6	2.7	7.5
		W	906–940	94.9	51.5	47.7	35.8	38.5	15.9	1.9	6.7	4.1	4.6
	West	H	153–158	98.1	54.2	57.3	31.4	29.2	1.9	0.6	12.7	4.4	18.4
		W	396–407	95.8	61.3	60.4	33.8	20.6	11.9	0.5	13.5	14.3	8.1

**Fig 4:**
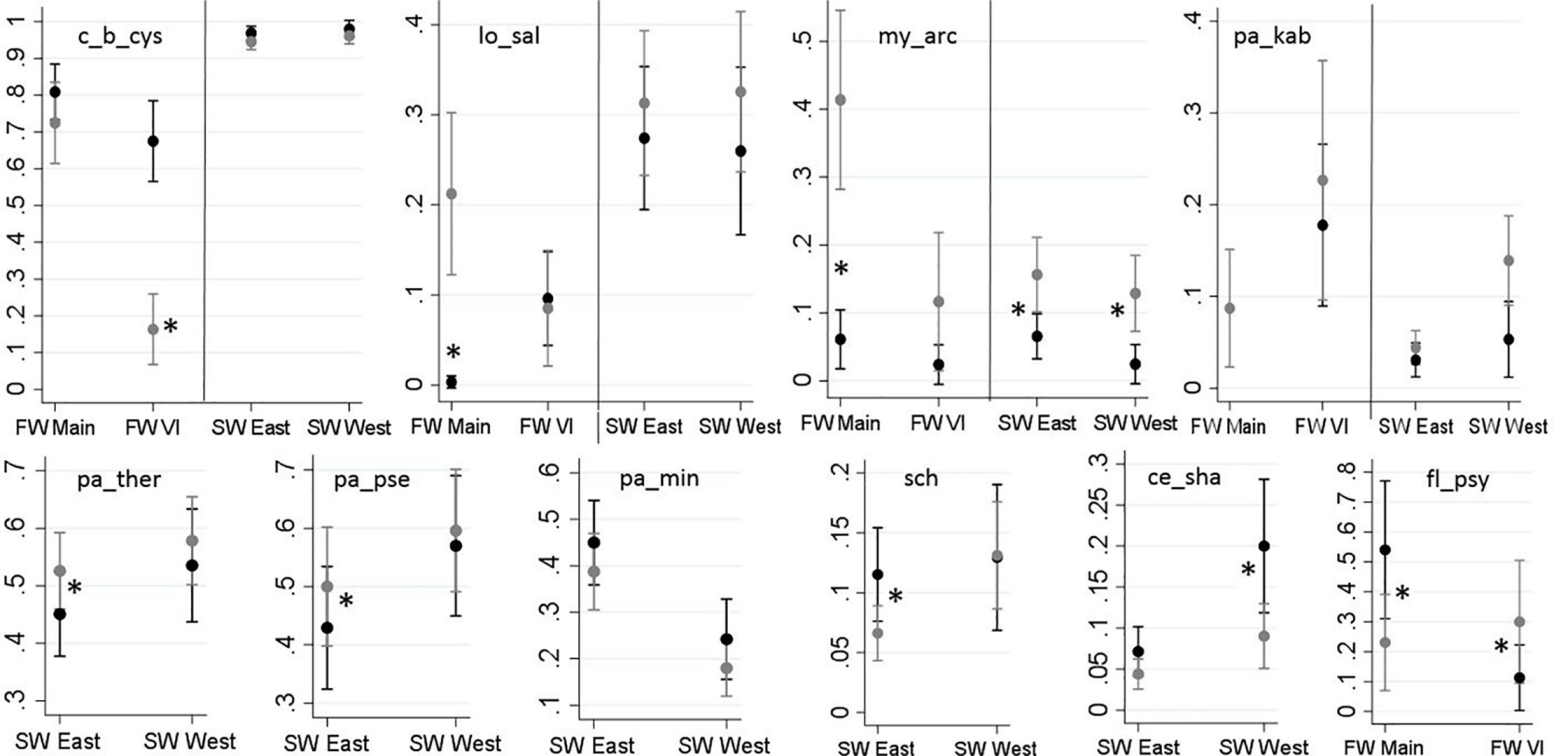
**Predicted probabilities of the detection of the prevalent infectious agents (Y axes) in 2,655 juvenile Coho salmon by sampling region (X axes) and origin (black: hatchery and grey: wild), based on logistic regression models. Sampling regions: 1) freshwater-Mainland; 2) freshwater-Vancouver Island (VI); 3) saltwater-east coast of VI; and 4) saltwater-west coast of VI. Note that the axes of these graphs are scaled to maximize resolution between hatchery and wild fish.** *Significant statistical comparisons (Bonferroni groups) for predicted probability of detection between hatchery and wild, within each category of region, are indicated with asterisks (P < 0.05).

Fig 4 summarizes the results of the logistic regression models for the common agents. In this Fig, relationships of the independent variables of interest (i.e. region and origin, while controlling for the random effects of year) with the predicted probability of infection for any given agent are illustrated. The corresponding table of results is included in the supportive information for reference (S3 Table). A brief description of changes in the prevalence of each common agent with respect to the fish origin is presented below.

The bacterial agent Ca. *B*. *cysticola* was detected in 89.3% of the samples. As reported in Table 3, its prevalence in marine waters (94.9–98.1%) is substantially higher than in freshwater (20.9–78.7%). The only statistically significant difference between hatchery and wild fish was observed on VI, where prevalence was higher in hatchery fish (P < 0.001) (Fig 4).

The microsporidian parasite, *L*. *salmonae*, was detected in 35% of fish in the marine environment, approximately 3.5× higher than that in freshwater (10%). The only significant difference in the prevalence of this agent between hatchery and wild Coho was observed in freshwater samples from the Mainland BC, where prevalence was higher in wild fish (P < 0.001; Fig 4).

The freshwater myxozoan parasite, *M*. *arcticus*, was consistently more prevalent in wild fish than in hatchery fish in all regions (Fig 4), with statistical significance in all but the freshwater VI samples (P = 0.712). Similarly, the overall prevalence of the myxozoan, *P*. *kabatai*, was higher in wild fish (vs. hatchery fish) in all regions, but the differences were not statistically significant (Fig 4).

The bacterium, *F*. *psychrophilum*, was only detected in freshwater. Its prevalence in the freshwater Mainland BC samples was significantly higher in hatchery fish than in wild fish (P = 0.001) whereas the opposite was true for VI (P = 0.009) (Fig 4).

Among the marine agents, microsporidian *P*. *theridion* and myxozoan *P*. *pseudobranchiocola* showed similar patterns, with significantly higher prevalence in wild than hatchery fish in the marine environment on the east coast of VI (with P = 0.039 and P = 0.047, respectively) (Fig 4). Both agents had approximately 10% higher prevalence on the west coast of VI compared to the east coast of VI (Table 3).

The overall prevalence of myxozoan parasite, *Parvicapsula minibicornis*, was substantially higher (by 16.5%) in fish sampled in marine waters off the east coast of VI than the west coast of VI. There were no significant differences in the prevalence between hatchery and wild fish within each region (Fig 4).

The bacterium, gill chlamydia (sch), was detected at significantly higher prevalence in hatchery-origin fish than in wild fish only in the marine waters off the east coast of VI (P = 0.008; Fig 4). Prevalence off the west coast of VI was 4.5% higher than the east coast of VI (Table 3).

On the west coast of VI, the prevalence of *Ceratonova shasta* (ce_sha) was significantly higher in hatchery-origin fish than in wild fish (P = 0.005; Fig 4).

The results for all pairwise comparisons made between hatchery and wild fish in our study (based on all statistical models) are presented in the supportive information for reference (S4 Table).

## Discussion

The three decade-long decline of Coho salmon populations in Canada and the United States [3,4,28] has resulted in the recent listings of multiple populations as endangered or of concern. This issue puts increasing pressure on the scientific community to find the potential causes and identify viable mitigation measures to prevent the extirpation of Coho salmon from the southernmost end of its distribution. Our research is the first comprehensive survey of a broad range of infectious agents in Coho salmon, and provides a necessary baseline for follow-up studies on infectious agents that may cause diseases and adversely affect the survival and productivity of Coho salmon stocks. Importantly, our data did not support the hypothesis that hatchery fish carry higher diversity and burden of infectious agents than wild fish. While there were some significant differences in the prevalences of specific pathogens between hatchery and wild Coho salmon within different regions, most of these differences were not consistent across all regions. In agreement with our conclusions, Meyers (2005) in a review of hatchery practices in Alaska for controlling two indicator pathogens (infectious hematopoietic necrosis virus, IHNV, and *Renibacterium salmoninarum*) concluded that hatchery practices or hatchery-derived fish did not increase the pathogen levels or prevalences among sympatric wild salmonid stocks [29].

### Overall diversity and burden

Diversity and burden of infectious agents in juvenile Coho salmon were substantially higher in the marine environment than in freshwater, consistent with recent studies on juvenile Chinook and Sockeye salmon [22,24,30]. The infectious burden and diversity of detected agents did not show a consistent distinction between hatchery and wild fish in our study. Rather, while divergence was observed in freshwater (with higher levels in wild than hatchery fish), diversity and infectious burden converged in the marine environment. These finding were consistent with a recent study carried out by our team on juvenile Chinook salmon, whereby differences in the infectious profiles between wild and hatchery juvenile Chinook from the Cowichan River system gradually faded as fish converged in the marine environment and migrated along the coast of BC [30]. Herein, difference between hatchery and wild fish in freshwater were predominantly driven by three parasites, *L*. *salmonae*, *M*. *arcticus*, and *P*. *kabatai* (described below). The latter two are myxozoans that require an alternate invertebrate host to complete their life-cycles. Because many hatcheries in the study region utilize ground water rather than river water for rearing, these differences can be explained by exposure to natural sources of infection. The significantly higher prevalences of *L*. *salmonae* and *M*. *arcticus* in wild fish (compared to hatchery fish) in Mainland BC were mainly influenced by the spring of 2016 samples, with detections in 97% and 74% of wild fish, respectively, while the prevalences of these two agents in Mainland BC over all other years did not exceed 5% and 33%, respectively. Average spring air temperatures for 2016 were the warmest on record since 1948 in most parts of Mainland BC [31,32], which may have contributed to the high prevalence of these pathogens in wild fish. *L*. *salmonae* xenoma development is known to be temperature dependent, with permissive temperatures between 9 and 20 ^o^C [33,34]. Our analyses also showed that the variability between study years was highly significant. However, we were not able to evaluate the prevalence of infectious agents on a yearly basis due to the lack of freshwater samples from several years.

### Prevalent infectious agents

Distributions of the common agents in our study locations were quite variable on an agent-by-agent basis; hence, we briefly discuss each agent, individually.

*Loma salmonae* is a microsporidian pathogen of salmonid fish which can cause salmonid microsporidial gill disease, SMGD, in BC [35]. While *L*. *salmonae* has been detected from wild and farmed Chinook salmon at high levels of prevalence [22,36], it was rarely detected in juvenile migratory Sockeye and farmed Atlantic salmon in BC [24,36]. Coho salmon appears to be sensitive to the infection but less sensitive than Chinook [34]. As explained above, 2016 samples drove the high prevalence of this agent in wild fish from Mainland BC.

*M*. *arcticus* is a myxozoan parasite, endemic to BC, which we have detected across a range of Pacific salmon species [22,24,30]. *M*. *arcticus* is known to infect juvenile salmon in freshwater [37]. The prevalence of *M*. *arcticus* in wild fish was consistently higher than in hatchery fish in all study locations. This finding may be due to the primary use of ground water in hatcheries, which presumably would not carry its annelid intermediate host (*Stylodrilus heringianus*). However, some exposure could occur when hatcheries sporadically switch to natural river water for rearing. The substantially higher prevalence of this agent in Mainland BC is likely associated with the abundance of its intermediate host, especially in the Fraser River system [38]. While *M*. *arcticus* has been associated with abnormal swimming behavior in naturally infected smolts [39], it appears to cause negligible direct pathological effects [40].

*P*. *kabatai* was first detected from adult pink salmon in Quinsam River [41]. In this study, we detected this parasite at low prevalence (overall, 6.2%) from both freshwater and saltwater samples. The intermediate host for *P*. *kabatai* is unknown, but it must reside in freshwater given the detection of this parasite in freshwater. This agent has recently been detected across other salmon species [22,24,30,36]. *P*. *kabatai* has been associated with increased likelihood of predation of juvenile Sockeye salmon by *Rhinoceros Auklets* [7]. There is still no information on the potential pathogenic effects of this agent in Pacific salmon species, which demands further research.

Since its first report in BC salmon in 2014, Ca. *B*. *cysticola* has consistently been the most frequent infectious agent detected in Pacific salmon species in both fresh and saltwater habitats in BC, typically with a prevalence > 80% [7,22,24,30]. Our findings in juvenile Coho salmon are consistent with other Pacific salmon species; however, wild juveniles sampled in freshwater systems on VI showed significantly lower prevalence (21%) than in other locations. A possible explanation for this finding may be the reduced abundance of Ca. *B*. *cysticola* in VI natural habitats, or it could be due to the relatively low number of wild juveniles captured/tested from this location in our study (n = 67). This bacterium is also prevalent in Europe and is associated with proliferative gill inflammation in net pens, but its role in the development of the disease has not yet been established [42,43]. However, Wang (2018) recently showed that in juvenile Chinook salmon, Ca. *B*. *cysticola* was significantly associated with the elevation of inflammatory response in the gill transcriptome [23].

*P*. *theridion* is a microsporidian parasite carried and possibly transmitted by salmon lice in saltwater. This parasite was discovered in farmed Atlantic salmon in western Norway in 2008, and is considered a primary agent in cases with high mortality linked to proliferative gill disease in western Norway [44,45]. This parasite has consistently been detected with high prevalences in seawater from farmed and wild salmon species in BC [22,24,30,36]. It has been shown that hatchery juvenile Coho salmon are fairly resistant to salmon lice [46]. On average, hatchery-origin fish have larger size as compared to their wild counterparts at the time of release, which may explain why we observed lower prevalence of *P*. *theridion* in our hatchery Coho than in wild fish.

*P*. *pseudobranchiocola* is a marine myxozoan parasite which has been detected from other Pacific salmon species in BC, especially Chinook [22,24,30]. The parasite can infect the pseudobranchs of gill tissue and has been associated with gill disease and mortality in farmed salmon [47], as well as impacts on swim performance and visual acuity [48]. *P*. *pseudobranchicola* was shown to be associated with increased risk of predation of juvenile Sockeye salmon [7]. Although the known life cycles of parvicapsulids involve a polychaete alternate host, the life cycle of *P*. *pseudobranchicola* is still unknown [49,50]. This parasite was more prevalent in wild Coho samples than in hatchery-origin fish in Salish Sea. One possible explanation for this finding could be longer exposure of wild fish to Seatrout (*Salmo trutta*), which has been identified as a natural host for *P*. *pseudobranchicola* [50].

*P*. *minibicornis* is an endemic freshwater-transmitted myxozoan which has been observed in other Pacific salmon species [22,24,51]. This parasite was the only agent, among the common agents, that had a higher prevalence on the east coast of VI (Salish Sea) than the west coast. This was likely due to the high estuarine abundance of its alternate host, *Manayunkia speciosa*, around Salish Sea. *P*. *minibicornis* typically causes lesions in kidney tissue, but is also associated with branchitis, osmoregulatory dysfunction, and pre-mature mortality in returning Sockeye in the Fraser River system [52]. *P*. *minibicornis* has been linked with parasite-associated mortality in freshwater in returning Sockeye salmon [18,22]. Juvenile Sockeye salmon infection with *P*. *minibicornis* was linked with increased risk of predation by *Rhinoceros Auklets* in the marine environment [7], and associated pathological changes and differential expression of immune genes have been demonstrated in juvenile Chinook salmon sampled in the marine environment [23].

*C*. *shasta* is a highly virulent myxozoan parasite that is endemic in salmon-bearing rivers throughout the Pacific Northwest [53]. It causes ceratomyxosis, which is reportable to the Canadian Food Inspection Agency and the World Organisation for Animal Health. The alternate oligochaete host (*Manayunkia speciosa*) resides in freshwater and estuarine environments. *C*. *shasta* can cause disease in all salmonid fish [54]. Interestingly, while this parasite is only known to be transmitted in freshwater (which is also the only environment in which pathogenicity has been established), our studies have repeatedly observed increasing abundances of this agent in the marine environment [22,24,30]. Higher prevalence of this agent in hatchery fish (compared to sympatric wild fish) in our study was in agreement with findings in Cowichan juvenile Chinook [30]. Given the higher abundance and larger difference between hatchery and wild fish on the west coast, we suspect that an abundance of hatchery fish from the Columbia River could explain this difference, as we know that *C*. *shasta* is highly prevalent within the Columbia River watershed [55,56]. With respect to the importance of this agent in saltwater, follow-up studies are necessary to elaborate on the pathogenicity and epidemiology of *C*. *shasta* in Pacific salmon species although there is some evidence of associated pathogenicity in juvenile Chinook salmon sampled in the ocean [23].

Salmon gill chlamydia (sch) was originally detected in farmed Atlantic salmon suffering from proliferative gill disease in Europe [57]. This agent has also been detected from other Pacific salmon species at the similar low to moderate levels of prevalence as in our study [22,24,30,36]. This bacterial agent was more prevalent in hatchery fish than wild fish, but only on the east coast of Vancouver Island. However, this trend was not consistent over the study years. We could not find any reasonable explanation for this finding. There is a clear knowledge gap around the pathogenesis and exact role of gill chlamydia in proliferative gill disease, which demands additional research.

*F*. *psychrophilum* is the causative agent of bacterial cold water disease in salmonid fish in freshwater [58]. The prevalence of *F*. *psychrophilum* in juvenile Chinook and Sockeye salmon in BC has been low (<5%) [22,24,30]. In agreement with the higher prevalence of this agent in our study (8.3%), it has been suggested that Coho salmon are particularly susceptible to the infection, which can lead to high levels of mortality, especially in juveniles [58]. Hatchery Coho from the Mainland BC showed substantially higher prevalence of *F*. *psychrophilum* compared to wild and VI fish in this study. It appeared that this difference was mostly driven by one Mainland hatchery, Chilliwack River Hatchery, experiencing very high prevalence of this bacterium in the two years of hatchery sampling (2013 and 2014), with detection in 50 out of the 59 samples tested.

### Low-prevalent agents

Low-prevalent infectious agents were included in the calculations and analyses of the overall diversity and infection burden. We did not find any prominent differences in the prevalence of these agents between hatchery and wild fish; nonetheless, we recognize that our power to detect differences for these agents was low. *Tetracapsuloides bryosalmonae*, *Ichthyophonus hoferi*, *Tenacibaculum maritimum*, and *Sphaerothecum destructuens* are all known pathogens in salmonid fish which can cause acute diseases with high levels of morbidity and mortality [7,59]. As a result, these agents may not be tolerated in high abundance, consistent with their observed low prevalences.

Erythrocytic necrosis virus (ENV) is an iridovirus that causes viral erythrocytic necrosis [60] in multiple species [61–63]. ENV is more prevalent in juvenile Sockeye and Chinook salmon [22,24]. It can cause anemia, reduction in stamina, and predispose fish to other infections, and/or increase the impact of other stressors (e.g. low oxygen) and predation, at times leading to population-level impacts in susceptible species [64]. Piscine orthoreovirus subtype-1 (PRV-1) is the causative agent of heart and skeletal muscle inflammation (HSMI) in farmed Atlantic salmon in Norway [65]. PRV-2 is the probable cause of erythrocytic inclusion body syndrome (EIBS) in Coho salmon in Japan [66]. Thus far, only PRV-1 has been detected in BC. Although PRV-1 has been associated with jaundice syndrome in farmed Chinook salmon [67], it has not yet been established as the causative agent of a disease in BC Pacific salmon [68,69].

All of the low prevalent infectious agents seem to be naturally occurring components of freshwater and marine ecosystems. Most of these agents have not been studied in Coho salmon at all and future studies are required to elucidate their potential pathogenic effects and association with survival and productivity of Coho salmon at the population level. In addition, we detected some other important pathogens of salmonids from our samples at prevalences <1%, such as viral hemorrhagic septicemia virus (VHSV) and *Renibacterium salmoninarum*. However, we were not able to assess the distribution of these agents due to very small number of detections; therefore, they stayed beyond the scope of this document. Our future studies will take aim at the most significant pathogens in Coho salmon.

In our recent studies on various Pacific salmon species in BC [7,22,24,30,36], some of the most virulent viral agents that have greatly affected farmed salmonids across the world; e.g., infectious salmon anemia virus (ISAV), infectious pancreatic necrosis virus (IPNV), salmonid alphavirus (SAV), and *Oncorynchus masou* herpesvirus (OMV) were not detected in any samples; therefore, we did not include those in our test panel. Infectious hematopoietic necrosis virus (IHNV) is a very important pathogen in salmonid fish, reportable to the Canadian Food Inspection Agency and the World Organisation for Animal Health. We did not detect this virus from our samples at all. Although IHNV has been detected in adult Coho salmon, it is believed that Coho are not susceptible to the disease [70,71].

### Limitations

There were a number of limitations in our study, including: (1) the uncertainty around the origin of a number of captured fish. As explained in the materials and methods section, there was a chance that the hatchery marks (fin clips and/or coded wire tags) were not detectable in hatchery-origin fish and they were misclassified as wild fish. In other words, all fish classified as ‘hatchery’ are definitely hatchery fish, but not all ‘wild fish’ were born wild. Therefore, we are more confident in the hatchery results than in wild. However, we believe this issue would have been limited to a small proportion of fish and would not likely bias our general conclusions; (2) some fish captured off the west coast may have originated from the Columbia River system because individual stock identification in Coho salmon was not possible in our study; (3) sampling from freshwater was not conducted in all study years. For instance, all 254 samples from hatcheries located in Mainland BC were collected in 2013 and 2014, while there were only 5 comparable wild samples available (from this region in 2014). This issue might have decreased the comparability of our freshwater samples (i.e. lack/shortage of appropriate matches); (4) our screening tool, Fluidigm BioMark^TM^ microfluidics qPCR, was used with some limitations (outlined under the laboratory analysis section), which demand caution in the interpretation of the results. For instance, there was a chance to misclassify a positive sample as ‘negative’ and therefore slightly underestimated the prevalence of an infectious agent when samples with very low RNA copy numbers for that agent were present. The loads of detected infectious agents for hatchery and wild fish, by study region, have been presented in the supplementary materials (S1 and S2 Figs). As shown, general patterns of the loads are consistent with the observed prevalences of common infectious agents.

Although we included random effects of years in our models, future comparisons between hatchery and wild fish in freshwater within each specific year may explain some of the infectious patterns observed in our study.

Although fish size has been shown to be variable among juvenile Coho salmon in freshwater, based on their origin [72], we did not include ‘size’ (with 160 missing values) in our models because its potential effect was partially captured by the origin of fish (hatchery versus wild). Perhaps, one of the reasons for higher burden and diversity of infectious agents observed in Mainland BC wild fish (compared to their hatchery counterparts) may be their greater susceptibility to some infectious agents due to their overall smaller size [72,73], but the absence of difference between hatchery and wild fish on Vancouver Island contradicts this possibility.

Despite the outlined limitations, we believe our large sample size, collected over an expansive number of years (i.e. 11 years) and over a wide geographical region in BC provides valuable baseline information on the presence and overall distribution of important infectious agents in juvenile Coho salmon, as well as reasonably robust comparisons between hatchery-origin and wild fish.

### Conclusions

This study provides a baseline dataset on the detection of infectious agents in wild and hatchery-reared Coho salmon, spanning a decade of sampling efforts. Our study does not support the hypothesis that hatchery fish carry higher diversity and burden of infectious agents compared to sympatric wild salmon species [15–17]. Overall, it appears that wild juvenile Coho from Mainland BC (mostly, originated from Fraser River system) carry higher levels of infections than their hatchery-origin counterparts, largely owing to parasites with alternate invertebrate hosts. While there were some differences between hatchery-origin and wild fish in the marine environment, they were rarely consistent between the east and west coast, suggesting that divergent distributions were not due to differences in susceptibility, but rather potentially explained by differences in habitat use along the coast, stock representation (e.g. Columbia-origin hatchery fish), and representation of hatchery and wild fish in different years. While our study sheds new light on the range of agents that Coho salmon carry, there remains a lot of unknowns regarding which agents have the highest pathogenic potential. Our future research will focus on histopathological evidence of diseases associated with these agents, shifts in agent distributions between seasons, and evaluation of potential associations between infections and marine survival and productivity of major Coho salmon populations.

## Supporting information

S1 Fig Distribution of the loads (log10 (copy number + 1)) of the prevalent infectious agents detected in 2,655 juvenile Coho salmon by sampling region and origin (hatchery: black & wild: white). Sampling regions: 1) freshwater-mainland; 2) freshwater-Vancouver Island (VI); 3) saltwater-east coast of VI; and 4) saltwater-west coast of VI. For infectious agent’s complete name, refer to Table 1.
(TIF)

S2 Fig Distribution of the loads (log10 (copy number + 1)) of the low-prevalent infectious agents (1% < prevalence < 5%) detected in 2,655 juvenile Coho salmon by sampling region and origin (hatchery: black & wild: white). Sampling regions: 1) freshwater-mainland; 2) freshwater-Vancouver Island (VI); 3) saltwater-east coast of VI; and 4) saltwater-west coast of VI. For infectious agent’s complete name, refer to Table 1.
(TIF)

S1 Table Location (M: Mainland and VI: Vancouver Island) and the number of fish (N) from the 13 freshwater hatcheries in the study. (PDF)

S2 Table Prevalence (%) and the range of samples tested (N) for the low-prevalent infectious agents (1% < prevalence < 5%) from 2,655 juvenile Coho salmon, by sampling region and origin (hatchery or wild). Four sampling regions: 1) freshwater-Mainland; 2) freshwater-Vancouver Island (VI); 3) saltwater-east coast of VI; and 4) saltwater-west coast of VI. For infectious agents’ complete names, refer to key in Table 1.
(PDF)

S3 Table Results of the logistic regression models, evaluating the associations between sampling region and origin, and the prevalence of each common infectious agent (dichotomous outcome). Sampling regions: 1) freshwater-Mainland; 2) freshwater-Vancouver Island (VI); 3) saltwater-east coast of VI; and 4) saltwater-west coast of VI.
(PDF)

S4 Table Pairwise comparisons for all of the models in the study: Diversity, Relative Infection Burdon (RIB-log), and the prevalence of ten common agents. Bonferroni adjustment has been applied to all analyses, including combinations of fish origin (Hatchery or Wild) and sampling regions: 1) freshwater-Mainland (FW Main); 2) freshwater-Vancouver Island (FW VI); 3) saltwater-east coast of VI (SW East); and 4) saltwater-west coast of VI (SW West).
(PDF)
