# Supplementary figures and images for: Comparison of infectious agents detected from hatchery and wild juvenile Coho salmon in British Columbia, 2008-2018
Source: PLoS One. 2019 Sep 3;14(9):e0221956. doi: 10.1371/journal.pone.0221956 (PMC6719873; doi:10.1371/journal.pone.0221956)

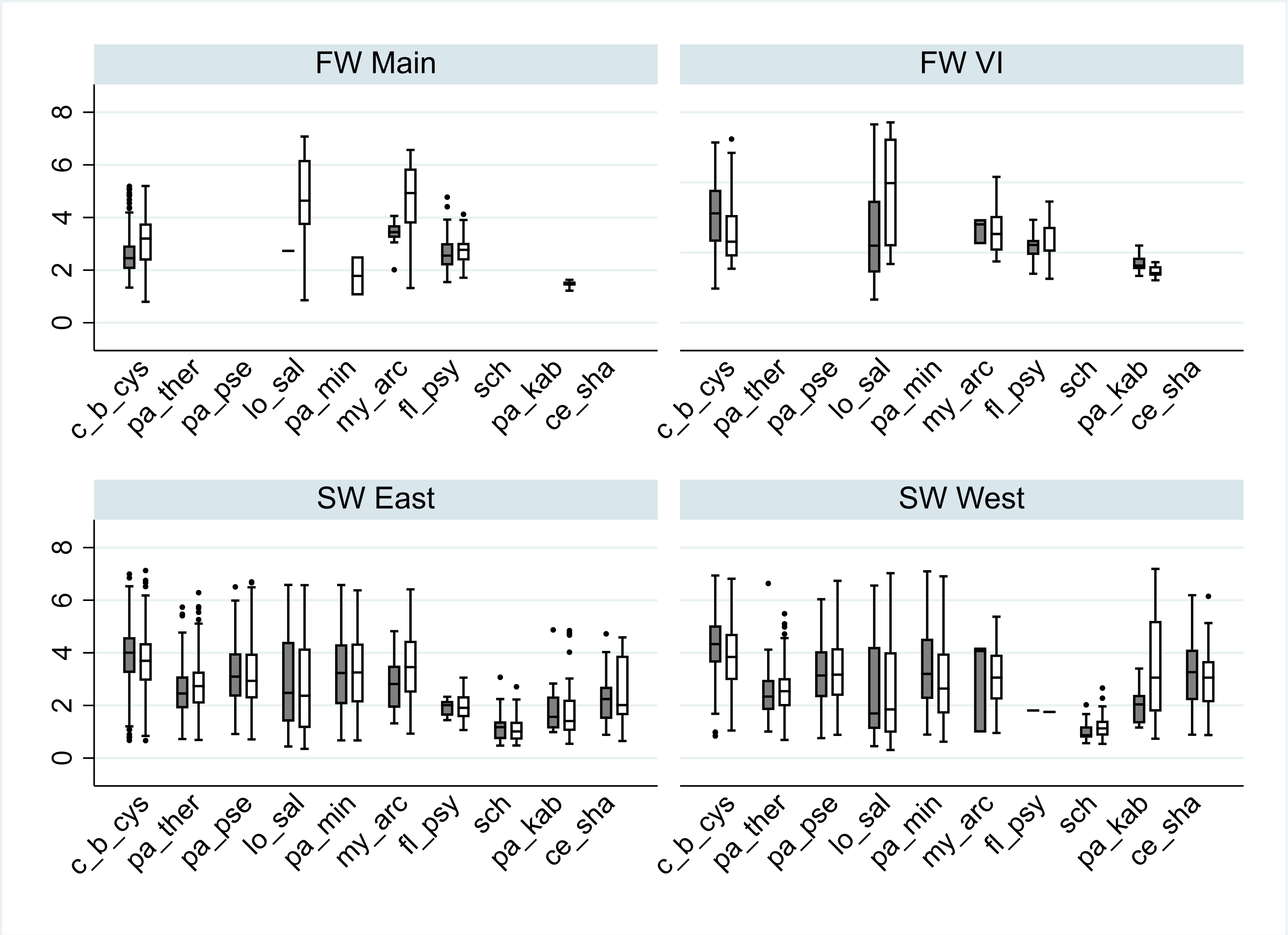

Supplement: S1 Fig — Sampling regions: 1) freshwater-mainland; 2) freshwater-Vancouver Island (VI); 3) saltwater-east coast of VI; and 4) saltwater-west coast of VI. For infectious agent’s complete name, refer to Table 1. (TIF) [file pone.0221956.s001.tif]

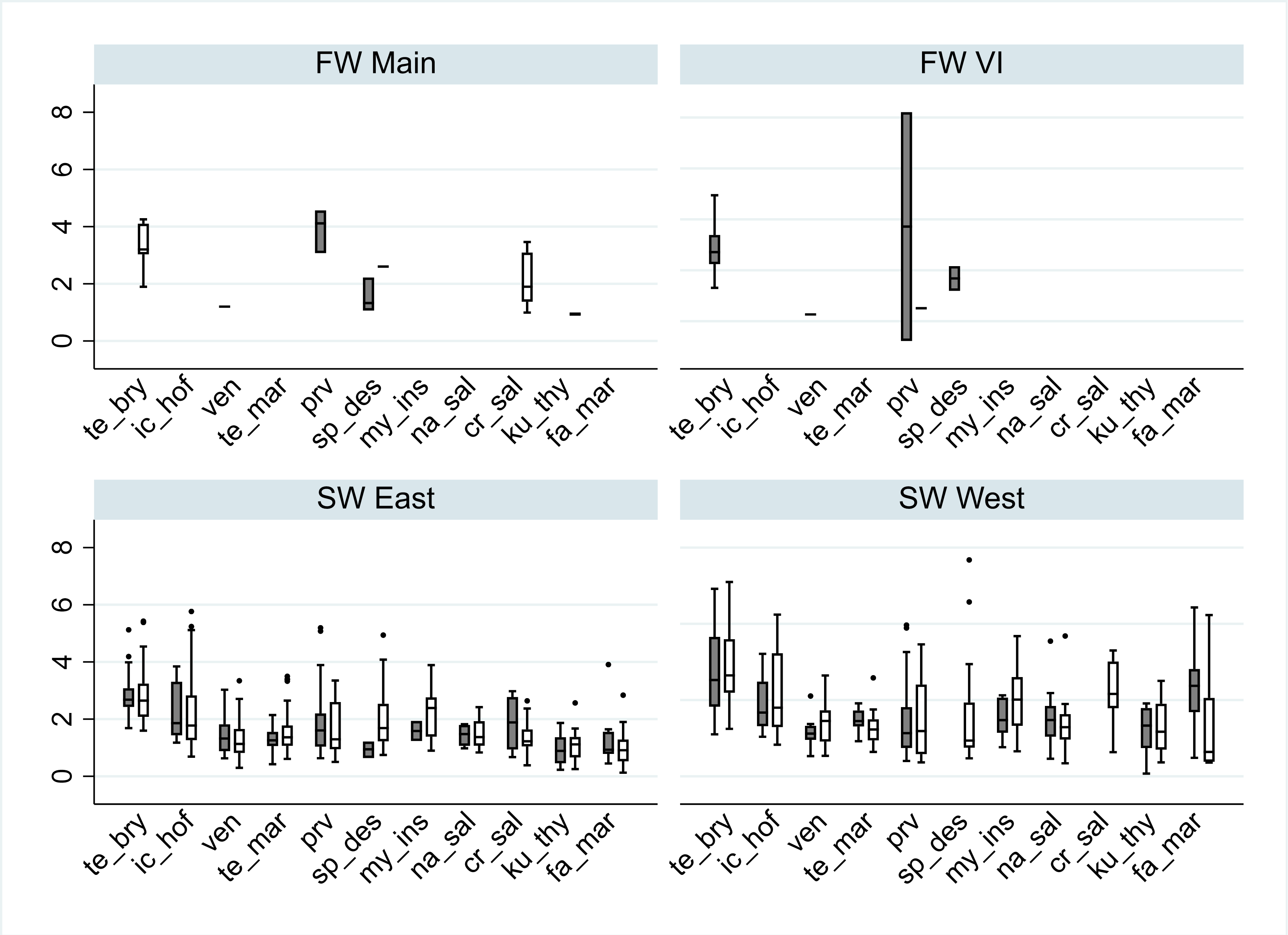

Supplement: S2 Fig — Sampling regions: 1) freshwater-mainland; 2) freshwater-Vancouver Island (VI); 3) saltwater-east coast of VI; and 4) saltwater-west coast of VI. For infectious agent’s complete name, refer to Table 1. (TIF) [file pone.0221956.s002.tif]
