# Supplementary material for: Comparison of infectious agents detected from hatchery and wild juvenile Coho salmon in British Columbia, 2008-2018
Source: PLoS One. 2019 Sep 3;14(9):e0221956. doi: 10.1371/journal.pone.0221956 (PMC6719873; doi:10.1371/journal.pone.0221956)
Supplement: S1 Table — (PDF) [file pone.0221956.s003.pdf]

S1 Table

S1 Table. Location (M: Mainland and VI: Vancouver Island) and the number of fish (N) from the 13 freshwater hatcheries in the study.

Hatchery	Location	N
Chilliwack River	M	59
Capilano River	M	46
Snootli Creek	M	38
Tenderfoot Creek	M	35
Chehalis River	M	28
Inch Creek	M	23
Spius Creek	M	5
Conuma River	VI	57
Puntledge River	VI	40
Robertson Creek	VI	30
Nitinat River	VI	29
Quinsam River	VI	26
Kitimat River	VI	20
Total	-	436
