# Supplementary material for: Comparison of infectious agents detected from hatchery and wild juvenile Coho salmon in British Columbia, 2008-2018
Source: PLoS One. 2019 Sep 3;14(9):e0221956. doi: 10.1371/journal.pone.0221956 (PMC6719873; doi:10.1371/journal.pone.0221956)
Supplement: S2 Table — Four sampling regions: 1) freshwater-Mainland; 2) freshwater-Vancouver Island (VI); 3) saltwater-east coast of VI; and 4) saltwater-west coast of VI. For infectious agents’ complete names, refer to key in Table 1. (PDF) [file pone.0221956.s004.pdf]

S2 Table

S2 Table. Prevalence (%) and the range of samples tested (N) for the low-prevalent infectious agents (1% < prevalence < 5%) from 2,655 juvenile Coho salmon, by sampling region and origin (hatchery or wild). Four sampling regions: 1) freshwater-mainland; 2) freshwater-Vancouver Island (VI); 3) saltwater-east coast of VI; and 4) saltwater-west coast of VI. For infectious agents' complete names, refer to key in Table 1.

	Region	Origin	N	te_bry	ic_hof	env	te_mar	prv	sp_des	my_ins	na_sal	cr_sal	ku_thy	fa_mar
FW	Main	H	254	0.00	0.00	0.39	0.00	1.18	1.18	0.00	0.00	0.00	0.00	0.00
		W	120-122	9.02	0.00	0.00	0.00	0.00	0.82	0.00	0.00	9.02	1.67	0.00
	VI	H	180-182	3.30	0.00	0.56	0.00	1.10	1.10	0.00	0.00	0.00	0.00	0.00
		W	65-67	0.00	0.00	0.00	0.00	1.49	0.00	0.00	0.00	0.00	0.00	0.00
SW	East	H	513-522	7.50	2.88	3.47	4.68	8.05	0.57	0.38	0.96	0.77	2.30	1.55
		W	927-942	4.15	5.86	4.15	3.67	0.74	2.34	0.85	0.42	2.23	2.34	1.51
	West	H	153-158	6.37	2.53	5.06	7.84	10.90	0.00	5.06	10.76	0.00	3.18	3.82
		W	402-408	5.90	5.69	5.94	3.71	1.72	5.90	7.65	5.41	1.72	0.98	1.99
