# Supplementary material for: Comparison of infectious agents detected from hatchery and wild juvenile Coho salmon in British Columbia, 2008-2018
Source: PLoS One. 2019 Sep 3;14(9):e0221956. doi: 10.1371/journal.pone.0221956 (PMC6719873; doi:10.1371/journal.pone.0221956)
Supplement: S3 Table — Sampling regions: 1) freshwater-Mainland; 2) freshwater-Vancouver Island (VI); 3) saltwater-east coast of VI; and 4) saltwater-west coast of VI. (PDF) [file pone.0221956.s005.pdf]

S3 Table. Results of the logistic regression models, evaluating the associations between sampling region and origin, and the prevalence of each common infectious agent (dichotomous outcome). Sampling regions: 1) freshwater-mainland; 2) freshwater-Vancouver Island (VI); 3) saltwater-east coast of VI; and 4) saltwater-west coast of VI.

**Agent**	**Variables**	**Coefficient**	**SE**	**95% CI**		**P-value**
				L	U	
**c_b_cys**	FW Main#Hatchery	Ref.[a]				
	FW Main#Wild	-0.49	0.34	-1.16	0.17	0.145
	FW VI#Hatchery	-0.74	0.24	-1.20	-0.28	0.002
	FW VI#Wild	-3.21	0.38	-3.97	-2.46	<0.001
	SW East#Hatchery	2.03	0.32	1.39	2.66	<0.001
	SW East#Wild	1.44	0.24	0.97	1.91	<0.001
	SW West#Hatchery	2.47	0.62	1.26	3.68	<0.001
	SW West#Wild	1.80	0.34	1.14	2.47	<0.001
**pa_ther**	SW East#Hatchery	Ref.				
	SW East#Wild	0.31	0.11	0.09	0.54	0.006
	SW West#Hatchery	0.35	0.20	-0.03	0.74	0.074
	SW West#Wild	0.53	0.16	0.23	0.84	0.001
**pa_pse**	SW East#Hatchery	Ref.				
	SW East#Wild	0.32	0.12	0.08	0.55	0.008
	SW West#Hatchery	0.63	0.20	0.24	1.03	0.002
	SW West#Wild	0.75	0.16	0.44	1.07	<0.001
**lo_sal**	FW Main#Hatchery	Ref.				
	FW Main#Wild	4.47	1.03	2.45	6.49	<0.001
	FW VI#Hatchery	3.50	1.03	1.48	5.51	0.001
	FW VI#Wild	3.36	1.07	1.25	5.47	0.002
	SW East#Hatchery	4.83	1.01	2.86	6.81	<0.001
	SW East#Wild	5.03	1.01	3.06	7.01	<0.001
	SW West#Hatchery	4.75	1.02	2.76	6.75	<0.001
	SW West#Wild	5.10	1.01	3.11	7.08	<0.001
**pa_min**	SW East#Hatchery	Ref.				
	SW East#Wild	-0.28	0.12	-0.51	-0.04	0.021
	SW West#Hatchery	-1.00	0.21	-1.42	-0.59	<0.001
	SW West#Wild	-1.40	0.17	-1.74	-1.06	<0.001
	FW Main#Hatchery	Ref.				
**my_arc**	FW Main#Wild	2.53	0.42	1.71	3.35	<0.001
	FW VI#Hatchery	-0.99	0.67	-2.30	0.31	0.136
	FW VI#Wild	0.73	0.57	-0.39	1.86	0.201
	SW East#Hatchery	0.08	0.38	-0.66	0.82	0.838
	SW East#Wild	1.09	0.34	0.42	1.76	0.001
	SW West#Hatchery	-0.97	0.67	-2.29	0.35	0.149
	SW West#Wild	0.85	0.37	0.12	1.59	0.023
**fl_psy**	FW Main#Hatchery	Ref.				
	FW Main#Wild	-1.78	0.48	-2.73	-0.84	<0.001
	FW VI#Hatchery	-2.83	0.32	-3.45	-2.21	<0.001
	FW VI#Wild	-1.33	0.45	-2.20	-0.45	0.003
**sch**	SW East#Hatchery	Ref.				
	SW East#Wild	-0.62	0.19	-0.99	-0.24	0.001
	SW West#Hatchery	0.13	0.29	-0.44	0.71	0.648
	SW West#Wild	0.15	0.23	-0.29	0.59	0.507
**pa_kab**	FW Main#Hatchery	Ref.				
	FW Main#Hatchery	0.00	(empty)			
	FW Main#Wild	-0.54	0.43	-1.38	0.31	0.212
	FW VI#Hatchery	0.30	0.32	-0.33	0.93	0.355
	FW VI#Wild	0.61	0.41	-0.18	1.41	0.132
	SW East#Hatchery	-1.65	0.33	-2.30	-1.00	<0.001
	SW East#Wild	-1.27	0.24	-1.74	-0.80	<0.001

	SW West#Hatchery	-1.07	0.42	-1.89	-0.25	0.010
	SW West#Wild	0.00	(omitted)			
**ce_sha**	SW East#Hatchery	Ref.				
	SW East#Wild	-0.52	0.23	-0.97	-0.06	0.025
	SW West#Hatchery	1.21	0.29	0.63	1.78	<0.001
	SW West#Wild	0.26	0.28	-0.29	0.80	0.356

[a] Reference (or baseline) category/region; 95 confidence interval for the coefficients indicate the comparison of individual region-origin with the baseline for each agent.

Note: in all models, the overall effects of sampling region-origin on the prevalence of individual agents were highly significant (P < 0.001 for all).
