# Supplementary material for: Comparison of infectious agents detected from hatchery and wild juvenile Coho salmon in British Columbia, 2008-2018
Source: PLoS One. 2019 Sep 3;14(9):e0221956. doi: 10.1371/journal.pone.0221956 (PMC6719873; doi:10.1371/journal.pone.0221956)
Supplement: S4 Table — Bonferroni adjustment has been applied to all analyses, including combinations of fish origin (Hatchery or Wild) and sampling regions: 1) freshwater-Mainland (FW Main); 2) freshwater-Vancouver Island (FW VI); 3) saltwater-east coast of VI (SW East); and 4) saltwater-west coast of VI (SW West). (PDF) [file pone.0221956.s006.pdf]

S4 Table. Pairwise comparisons for all of the models in the study: Diversity, Relative Infection Burdon (RIB-log), and the prevalence of ten common agents. Bonferroni adjustment has been applied to all analyses, including combinations of fish origin (Hatchery or Wild) and sampling regions: 1) freshwater-mainland (FW Main); 2) freshwater-Vancouver Island (FW VI); 3) saltwater-east coast of VI (SW East); and 4) saltwater-west coast of VI (SW West).

			**95% CI**		
**Model**	Coefficient	SE	L	U	P-value
**Diversity**					
**(FW Main#Wild) vs (FW Main#Hatchery)**	0.386	0.088	0.111	0.661	<0.001
**(FW VI#Hatchery) vs (FW Main#Hatchery)**	-0.171	0.089	-0.448	0.107	1.000
**(FW VI#Wild) vs (FW Main#Hatchery)**	-0.325	0.138	-0.755	0.105	0.510
**(SW East#Hatchery) vs (FW Main#Hatchery)**	0.880	0.061	0.689	1.072	<0.001
**(SW East#Wild) vs (FW Main#Hatchery)**	0.912	0.059	0.727	1.097	<0.001
**(SW West#Hatchery) vs (FW Main#Hatchery)**	0.998	0.071	0.777	1.219	<0.001
**(SW West#Wild) vs (FW Main#Hatchery)**	1.008	0.064	0.809	1.207	<0.001
**(FW VI#Hatchery) vs (FW Main#Wild)**	-0.557	0.098	-0.862	-0.252	<0.001
**(FW VI#Wild) vs (FW Main#Wild)**	-0.711	0.142	-1.155	-0.267	<0.001
**(SW East#Hatchery) vs (FW Main#Wild)**	0.494	0.071	0.272	0.716	<0.001
**(SW East#Wild) vs (FW Main#Wild)**	0.526	0.069	0.309	0.743	<0.001
**(SW West#Hatchery) vs (FW Main#Wild)**	0.612	0.078	0.367	0.857	<0.001
**(SW West#Wild) vs (FW Main#Wild)**	0.622	0.072	0.398	0.846	<0.001
**(FW VI#Wild) vs (FW VI#Hatchery)**	-0.154	0.145	-0.609	0.300	1.000
**(SW East#Hatchery) vs (FW VI#Hatchery)**	1.051	0.076	0.812	1.290	<0.001
**(SW East#Wild) vs (FW VI#Hatchery)**	1.082	0.075	0.850	1.315	<0.001
**(SW West#Hatchery) vs (FW VI#Hatchery)**	1.169	0.083	0.911	1.427	<0.001
**(SW West#Wild) vs (FW VI#Hatchery)**	1.179	0.077	0.939	1.419	<0.001
**(SW East#Hatchery) vs (FW VI#Wild)**	1.205	0.128	0.804	1.606	<0.001
**(SW East#Wild) vs (FW VI#Wild)**	1.237	0.127	0.839	1.634	<0.001
**(SW West#Hatchery) vs (FW VI#Wild)**	1.323	0.133	0.908	1.738	<0.001
**(SW West#Wild) vs (FW VI#Wild)**	1.333	0.129	0.928	1.737	<0.001
**(SW East#Wild) vs (SW East#Hatchery)**	0.032	0.031	-0.065	0.128	1.000
**(SW West#Hatchery) vs (SW East#Hatchery)**	0.118	0.050	-0.039	0.275	0.532
**(SW West#Wild) vs (SW East#Hatchery)**	0.128	0.039	0.006	0.250	0.029
**(SW West#Hatchery) vs (SW East#Wild)**	0.086	0.047	-0.060	0.232	1.000
**(SW West#Wild) vs (SW East#Wild)**	0.096	0.034	-0.011	0.204	0.142
**(SW West#Wild) vs (SW West#Hatchery)**	0.010	0.050	-0.145	0.165	1.000
**RIB-log**					
**(FW Main#Wild) vs (FW Main#Hatchery)**	0.444	0.117	0.078	0.810	0.004
**(FW VI#Hatchery) vs (FW Main#Hatchery)**	-0.631	0.097	-0.935	-0.328	<0.001
**(FW VI#Wild) vs (FW Main#Hatchery)**	-0.490	0.136	-0.916	-0.065	0.009
**(SW East#Hatchery) vs (FW Main#Hatchery)**	0.618	0.078	0.373	0.863	<0.001
**(SW East#Wild) vs (FW Main#Hatchery)**	0.533	0.074	0.303	0.763	<0.001
**(SW West#Hatchery) vs (FW Main#Hatchery)**	0.866	0.103	0.544	1.187	<0.001
**(SW West#Wild) vs (FW Main#Hatchery)**	0.767	0.086	0.497	1.037	<0.001
**(FW VI#Hatchery) vs (FW Main#Wild)**	-1.075	0.124	-1.462	-0.689	<0.001
**(FW VI#Wild) vs (FW Main#Wild)**	-0.934	0.152	-1.408	-0.460	<0.001
**(SW East#Hatchery) vs (FW Main#Wild)**	0.174	0.103	-0.147	0.495	1.000
**(SW East#Wild) vs (FW Main#Wild)**	0.089	0.099	-0.220	0.399	1.000
**(SW West#Hatchery) vs (FW Main#Wild)**	0.422	0.121	0.044	0.800	0.014
**(SW West#Wild) vs (FW Main#Wild)**	0.323	0.106	-0.009	0.656	0.067
**(FW VI#Wild) vs (FW VI#Hatchery)**	0.141	0.146	-0.314	0.596	1.000
**(SW East#Hatchery) vs (FW VI#Hatchery)**	1.249	0.092	0.963	1.536	<0.001
**(SW East#Wild) vs (FW VI#Hatchery)**	1.165	0.087	0.894	1.436	<0.001
**(SW West#Hatchery) vs (FW VI#Hatchery)**	1.497	0.110	1.152	1.842	<0.001
**(SW West#Wild) vs (FW VI#Hatchery)**	1.399	0.096	1.099	1.698	<0.001
**(SW East#Hatchery) vs (FW VI#Wild)**	1.108	0.128	0.709	1.508	<0.001
**(SW East#Wild) vs (FW VI#Wild)**	1.024	0.125	0.635	1.413	<0.001

**(SW West#Hatchery) vs (FW VI#Wild)**	1.356	0.143	0.909	1.803	<0.001
**(SW West#Wild) vs (FW VI#Wild)**	1.258	0.133	0.843	1.672	<0.001
**(SW East#Wild) vs (SW East#Hatchery)**	-0.085	0.053	-0.251	0.082	1.000
**(SW West#Hatchery) vs (SW East#Hatchery)**	0.248	0.091	-0.036	0.531	0.178
**(SW West#Wild) vs (SW East#Hatchery)**	0.149	0.070	-0.070	0.369	0.940
**(SW West#Hatchery) vs (SW East#Wild)**	0.332	0.085	0.067	0.598	0.003
**(SW West#Wild) vs (SW East#Wild)**	0.234	0.062	0.040	0.428	0.005
**(SW West#Wild) vs (SW West#Hatchery)**	-0.098	0.092	-0.385	0.188	1.000
**c_b_cys**					
**(FW Main#Wild) vs (FW Main#Hatchery)**	-0.493	0.339	-1.551	0.564	1.000
**(FW VI#Hatchery) vs (FW Main#Hatchery)**	-0.740	0.236	-1.477	-0.002	0.049
**(FW VI#Wild) vs (FW Main#Hatchery)**	-3.215	0.385	-4.417	-2.013	<0.001
**(SW East#Hatchery) vs (FW Main#Hatchery)**	2.027	0.323	1.019	3.036	<0.001
**(SW East#Wild) vs (FW Main#Hatchery)**	1.440	0.242	0.684	2.196	<0.001
**(SW West#Hatchery) vs (FW Main#Hatchery)**	2.469	0.617	0.541	4.396	0.002
**(SW West#Wild) vs (FW Main#Hatchery)**	1.802	0.339	0.742	2.862	<0.001
**(FW VI#Hatchery) vs (FW Main#Wild)**	-0.246	0.343	-1.318	0.825	1.000
**(FW VI#Wild) vs (FW Main#Wild)**	-2.722	0.409	-4.000	-1.443	<0.001
**(SW East#Hatchery) vs (FW Main#Wild)**	2.520	0.370	1.366	3.675	<0.001
**(SW East#Wild) vs (FW Main#Wild)**	1.933	0.305	0.979	2.887	<0.001
**(SW West#Hatchery) vs (FW Main#Wild)**	2.962	0.643	0.953	4.971	<0.001
**(SW West#Wild) vs (FW Main#Wild)**	2.295	0.378	1.113	3.477	<0.001
**(FW VI#Wild) vs (FW VI#Hatchery)**	-2.475	0.400	-3.725	-1.226	<0.001
**(SW East#Hatchery) vs (FW VI#Hatchery)**	2.767	0.337	1.714	3.820	<0.001
**(SW East#Wild) vs (FW VI#Hatchery)**	2.180	0.258	1.374	2.986	<0.001
**(SW West#Hatchery) vs (FW VI#Hatchery)**	3.208	0.619	1.275	5.141	<0.001
**(SW West#Wild) vs (FW VI#Hatchery)**	2.541	0.345	1.463	3.620	<0.001
**(SW East#Hatchery) vs (FW VI#Wild)**	5.242	0.432	3.894	6.590	<0.001
**(SW East#Wild) vs (FW VI#Wild)**	4.655	0.375	3.485	5.825	<0.001
**(SW West#Hatchery) vs (FW VI#Wild)**	5.684	0.680	3.560	7.808	<0.001
**(SW West#Wild) vs (FW VI#Wild)**	5.017	0.450	3.611	6.423	<0.001
**(SW East#Wild) vs (SW East#Hatchery)**	-0.587	0.304	-1.537	0.363	1.000
**(SW West#Hatchery) vs (SW East#Hatchery)**	0.442	0.648	-1.582	2.465	1.000
**(SW West#Wild) vs (SW East#Hatchery)**	-0.225	0.386	-1.432	0.981	1.000
**(SW West#Hatchery) vs (SW East#Wild)**	1.029	0.608	-0.871	2.929	1.000
**(SW West#Wild) vs (SW East#Wild)**	0.362	0.312	-0.614	1.337	1.000
**(SW West#Wild) vs (SW West#Hatchery)**	-0.667	0.639	-2.663	1.329	1.000
**lo_sal**					
**(FW Main#Wild) vs (FW Main#Hatchery)**	4.473	1.030	1.254	7.691	<0.001
**(FW VI#Hatchery) vs (FW Main#Hatchery)**	3.495	1.030	0.279	6.712	0.019
**(FW VI#Wild) vs (FW Main#Hatchery)**	3.360	1.075	0.003	6.717	0.049
**(SW East#Hatchery) vs (FW Main#Hatchery)**	4.833	1.009	1.682	7.984	<0.001
**(SW East#Wild) vs (FW Main#Hatchery)**	5.035	1.007	1.891	8.179	<0.001
**(SW West#Hatchery) vs (FW Main#Hatchery)**	4.755	1.020	1.568	7.942	<0.001
**(SW West#Wild) vs (FW Main#Hatchery)**	5.098	1.012	1.937	8.259	<0.001
**(FW VI#Hatchery) vs (FW Main#Wild)**	-0.977	0.334	-2.022	0.067	0.097
**(FW VI#Wild) vs (FW Main#Wild)**	-1.113	0.441	-2.491	0.265	0.326
**(SW East#Hatchery) vs (FW Main#Wild)**	0.360	0.243	-0.399	1.119	1.000
**(SW East#Wild) vs (FW Main#Wild)**	0.562	0.235	-0.172	1.296	0.469
**(SW West#Hatchery) vs (FW Main#Wild)**	0.282	0.285	-0.609	1.172	1.000
**(SW West#Wild) vs (FW Main#Wild)**	0.625	0.254	-0.168	1.418	0.387
**(FW VI#Wild) vs (FW VI#Hatchery)**	-0.135	0.458	-1.566	1.295	1.000
**(SW East#Hatchery) vs (FW VI#Hatchery)**	1.337	0.266	0.506	2.169	<0.001
**(SW East#Wild) vs (FW VI#Hatchery)**	1.539	0.256	0.739	2.340	<0.001
**(SW West#Hatchery) vs (FW VI#Hatchery)**	1.259	0.300	0.324	2.195	0.001
**(SW West#Wild) vs (FW VI#Hatchery)**	1.602	0.272	0.753	2.451	<0.001
**(SW East#Hatchery) vs (FW VI#Wild)**	1.473	0.396	0.235	2.710	0.006

**(SW East#Wild) vs (FW VI#Wild)**	1.675	0.390	0.457	2.892	<0.001
**(SW West#Hatchery) vs (FW VI#Wild)**	1.395	0.422	0.077	2.712	0.026
**(SW West#Wild) vs (FW VI#Wild)**	1.738	0.404	0.475	3.000	<0.001
**(SW East#Wild) vs (SW East#Hatchery)**	0.202	0.121	-0.177	0.581	1.000
**(SW West#Hatchery) vs (SW East#Hatchery)**	-0.078	0.209	-0.731	0.575	1.000
**(SW West#Wild) vs (SW East#Hatchery)**	0.265	0.161	-0.239	0.769	1.000
**(SW West#Hatchery) vs (SW East#Wild)**	-0.280	0.194	-0.887	0.327	1.000
**(SW West#Wild) vs (SW East#Wild)**	0.063	0.141	-0.377	0.503	1.000
**(SW West#Wild) vs (SW West#Hatchery)**	0.343	0.210	-0.314	1.000	1.000
**my_arc**					
**(FW Main#Wild) vs (FW Main#Hatchery)**	2.532	0.418	1.226	3.839	<0.001
**(FW VI#Hatchery) vs (FW Main#Hatchery)**	-0.994	0.667	-3.078	1.089	1.000
**(FW VI#Wild) vs (FW Main#Hatchery)**	0.734	0.575	-1.060	2.529	1.000
**(SW East#Hatchery) vs (FW Main#Hatchery)**	0.077	0.377	-1.101	1.255	1.000
**(SW East#Wild) vs (FW Main#Hatchery)**	1.091	0.342	0.024	2.158	0.039
**(SW West#Hatchery) vs (FW Main#Hatchery)**	-0.969	0.672	-3.068	1.129	1.000
**(SW West#Wild) vs (FW Main#Hatchery)**	0.853	0.375	-0.317	2.023	0.637
**(FW VI#Hatchery) vs (FW Main#Wild)**	-3.526	0.655	-5.571	-1.482	<0.001
**(FW VI#Wild) vs (FW Main#Wild)**	-1.798	0.529	-3.449	-0.147	0.019
**(SW East#Hatchery) vs (FW Main#Wild)**	-2.455	0.310	-3.424	-1.487	<0.001
**(SW East#Wild) vs (FW Main#Wild)**	-1.441	0.270	-2.284	-0.598	<0.001
**(SW West#Hatchery) vs (FW Main#Wild)**	-3.502	0.635	-5.484	-1.519	<0.001
**(SW West#Wild) vs (FW Main#Wild)**	-1.679	0.301	-2.620	-0.738	<0.001
**(FW VI#Wild) vs (FW VI#Hatchery)**	1.729	0.773	-0.688	4.145	0.712
**(SW East#Hatchery) vs (FW VI#Hatchery)**	1.071	0.633	-0.907	3.049	1.000
**(SW East#Wild) vs (FW VI#Hatchery)**	2.085	0.612	0.175	3.996	0.018
**(SW West#Hatchery) vs (FW VI#Hatchery)**	0.025	0.838	-2.593	2.642	1.000
**(SW West#Wild) vs (FW VI#Hatchery)**	1.847	0.627	-0.111	3.806	0.090
**(SW East#Hatchery) vs (FW VI#Wild)**	-0.657	0.515	-2.265	0.951	1.000
**(SW East#Wild) vs (FW VI#Wild)**	0.357	0.489	-1.171	1.885	1.000
**(SW West#Hatchery) vs (FW VI#Wild)**	-1.704	0.757	-4.069	0.662	0.686
**(SW West#Wild) vs (FW VI#Wild)**	0.119	0.514	-1.487	1.724	1.000
**(SW East#Wild) vs (SW East#Hatchery)**	1.014	0.207	0.366	1.662	<0.001
**(SW West#Hatchery) vs (SW East#Hatchery)**	-1.046	0.618	-2.978	0.886	1.000
**(SW West#Wild) vs (SW East#Hatchery)**	0.776	0.261	-0.040	1.593	0.084
**(SW West#Hatchery) vs (SW East#Wild)**	-2.061	0.595	-3.920	-0.201	0.015
**(SW West#Wild) vs (SW East#Wild)**	-0.238	0.199	-0.861	0.385	1.000
**(SW West#Wild) vs (SW West#Hatchery)**	1.822	0.606	-0.070	3.715	0.074
**pa_kab**					
**(FW VI#Hatchery) vs (FW Main#Wild)**	0.834	0.480	-0.667	2.335	1.000
**(FW VI#Wild) vs (FW Main#Wild)**	1.148	0.527	-0.497	2.793	0.819
**(SW East#Hatchery) vs (FW Main#Wild)**	-1.111	0.481	-2.613	0.391	0.583
**(SW East#Wild) vs (FW Main#Wild)**	-0.732	0.433	-2.085	0.621	1.000
**(SW West#Hatchery) vs (FW Main#Wild)**	-0.537	0.556	-2.273	1.199	1.000
**(SW West#Wild) vs (FW Main#Wild)**	0.536	0.430	-0.806	1.878	1.000
**(FW VI#Wild) vs (FW VI#Hatchery)**	0.314	0.459	-1.120	1.749	1.000
**(SW East#Hatchery) vs (FW VI#Hatchery)**	-1.945	0.393	-3.174	-0.716	<0.001
**(SW East#Wild) vs (FW VI#Hatchery)**	-1.566	0.328	-2.590	-0.542	<0.001
**(SW West#Hatchery) vs (FW VI#Hatchery)**	-1.371	0.470	-2.838	0.096	0.099
**(SW West#Wild) vs (FW VI#Hatchery)**	-0.298	0.322	-1.303	0.708	1.000
**(SW East#Hatchery) vs (FW VI#Wild)**	-2.259	0.442	-3.642	-0.877	<0.001
**(SW East#Wild) vs (FW VI#Wild)**	-1.880	0.391	-3.102	-0.658	<0.001
**(SW West#Hatchery) vs (FW VI#Wild)**	-1.685	0.531	-3.344	-0.026	0.042
**(SW West#Wild) vs (FW VI#Wild)**	-0.612	0.406	-1.881	0.657	1.000
**(SW East#Wild) vs (SW East#Hatchery)**	0.379	0.321	-0.624	1.382	1.000
**(SW West#Hatchery) vs (SW East#Hatchery)**	0.574	0.484	-0.938	2.087	1.000
**(SW West#Wild) vs (SW East#Hatchery)**	1.648	0.331	0.615	2.681	<0.001

**(SW West#Hatchery) vs (SW East#Wild)**	0.195	0.431	-1.150	1.540	1.000
**(SW West#Wild) vs (SW East#Wild)**	1.268	0.241	0.515	2.021	<0.001
**(SW West#Wild) vs (SW West#Hatchery)**	1.073	0.419	-0.235	2.382	0.291
**pa_ther**					
**(SW East#Wild) vs (SW East#Hatchery)**	0.313	0.115	0.010	0.616	0.039
**(SW West#Hatchery) vs (SW East#Hatchery)**	0.352	0.197	-0.168	0.871	0.444
**(SW West#Wild) vs (SW East#Hatchery)**	0.533	0.156	0.122	0.943	0.004
**(SW West#Hatchery) vs (SW East#Wild)**	0.039	0.184	-0.445	0.524	1.000
**(SW West#Wild) vs (SW East#Wild)**	0.220	0.139	-0.146	0.585	0.676
**(SW West#Wild) vs (SW West#Hatchery)**	0.181	0.198	-0.342	0.703	1.000
**pa_pse**					
**(SW East#Wild) vs (SW East#Hatchery)**	0.317	0.119	0.003	0.632	0.047
**(SW West#Hatchery) vs (SW East#Hatchery)**	0.632	0.202	0.100	1.163	0.010
**(SW West#Wild) vs (SW East#Hatchery)**	0.751	0.161	0.327	1.175	<0.001
**(SW West#Hatchery) vs (SW East#Wild)**	0.314	0.187	-0.178	0.807	0.554
**(SW West#Wild) vs (SW East#Wild)**	0.434	0.141	0.061	0.806	0.013
**(SW West#Wild) vs (SW West#Hatchery)**	0.119	0.202	-0.413	0.651	1.000
**pa_min**					
**(SW East#Wild) vs (SW East#Hatchery)**	-0.275	0.120	-0.591	0.040	0.128
**(SW West#Hatchery) vs (SW East#Hatchery)**	-1.002	0.212	-1.562	-0.442	<0.001
**(SW West#Wild) vs (SW East#Hatchery)**	-1.397	0.173	-1.854	-0.941	<0.001
**(SW West#Hatchery) vs (SW East#Wild)**	-0.727	0.198	-1.249	-0.204	0.001
**(SW West#Wild) vs (SW East#Wild)**	-1.122	0.156	-1.532	-0.712	<0.001
**(SW West#Wild) vs (SW West#Hatchery)**	-0.395	0.222	-0.980	0.189	0.447
**sch**					
**(SW East#Wild) vs (SW East#Hatchery)**	-0.616	0.192	-1.124	-0.109	0.008
**(SW West#Hatchery) vs (SW East#Hatchery)**	0.134	0.293	-0.639	0.907	1.000
**(SW West#Wild) vs (SW East#Hatchery)**	0.150	0.226	-0.447	0.748	1.000
**(SW West#Hatchery) vs (SW East#Wild)**	0.750	0.284	0.002	1.498	0.049
**(SW West#Wild) vs (SW East#Wild)**	0.767	0.214	0.203	1.331	0.002
**(SW West#Wild) vs (SW West#Hatchery)**	0.017	0.287	-0.739	0.772	1.000
**ce_sha**					
**(SW East#Wild) vs (SW East#Hatchery)**	-0.519	0.232	-1.132	0.094	0.152
**(SW West#Hatchery) vs (SW East#Hatchery)**	1.206	0.293	0.434	1.978	<0.001
**(SW West#Wild) vs (SW East#Hatchery)**	0.257	0.278	-0.477	0.991	1.000
**(SW West#Hatchery) vs (SW East#Wild)**	1.726	0.280	0.986	2.465	<0.001
**(SW West#Wild) vs (SW East#Wild)**	0.776	0.266	0.075	1.478	0.021
**(SW West#Wild) vs (SW West#Hatchery)**	-0.949	0.283	-1.697	-0.202	0.005
**fl_psy**					
**(FW Main#Wild) vs (FW Main#Hatchery)**	-1.784	0.483	-3.059	-0.510	0.001
**(FW VI#Hatchery) vs (FW Main#Hatchery)**	-2.834	0.316	-3.668	-2.000	<0.001
**(FW VI#Wild) vs (FW Main#Hatchery)**	-1.328	0.446	-2.505	-0.152	0.017
**(FW VI#Hatchery) vs (FW Main#Wild)**	-1.050	0.469	-2.286	0.187	0.151
**(FW VI#Wild) vs (FW Main#Wild)**	0.456	0.452	-0.736	1.648	1.000
**(FW VI#Wild) vs (FW VI#Hatchery)**	1.506	0.474	0.256	2.755	0.009
